# XChondNet: Explainable Chondrogenic Tumor Diagnosis by Spatial-Context-Aware Synergistic Deep Feature Fusion

**DOI:** 10.3390/s26175518

**Published:** 2026-08-31

**Authors:** Shuai Xiao, Yuefeng Xie, Guipeng Lan, Aidong Liu, Jiachen Yang

**Affiliations:** 1 School of Electrical and Information Engineering, Tianjin University, Tianjin 300072, China; xs611@tju.edu.cn (S.X.); xieyf@tju.edu.cn (Y.X.); yangjiachen@tju.edu.cn (J.Y.); 2 Department of Pathology, Tianjin Hospital, No. 406 Jiefang Nan Street, Hexi District, Tianjin 300211, China; liuaidong303@163.com

**Keywords:** feature fusion, chondrogenic tumors, digital pathology images, tumor diagnosis, parallel classifier mechanism

## Abstract

As a common benign bone tumor, the pathological diagnosis of chondrogenic tumors needs to accurately analyze the calcification pattern of the cartilage matrix, key spatial topology information, and other important indicators. However, chondrogenic tumors are a heterogeneous group of tumors and a rare disease: doctors lack sufficient reference data and experience in diagnosis. At the same time, complex spatial structural information also increases the difficulty of diagnosis, which leads to inconsistencies in the diagnosis’s results of different doctors. In contrast, with the development of technology, artificial intelligence (AI), with its fast, accurate, and robust characteristics, can effectively improve the efficiency and accuracy of diagnosis. However, the application of AI in this field is still unrecognized, so it is urgent to develop a model that can help doctors in diagnosis to improve accuracy and efficiency. In this study, we propose XChondNet, an explainable spatial-context-aware synergistic deep feature fusion model for WSI-based chondrogenic tumor classification. The model proposes a fusion mechanism of pathological and positional features so that the model can effectively perceive spatial structural information and a parallel classifier mechanism based on potential coding, which can effectively solve the problem of class imbalance in chondrogenic tumor data. We evaluated the XChondNet model on our chondrogenic tumor dataset and the experimental results verified its effectiveness in the classification of the chondrogenic tumor subtype. In the test phase, XChondNet consistently achieved superior performance across different feature extractors. Compared with two strong MIL baselines, DTFD-MIL and RRT-MIL, XChondNet improved the average ACC from 85.16% to 86.58% (1.42 percentage points), with statistically significant differences confirmed by a paired *t*-test ($p=2.61\times 10^{-5}$) and Wilcoxon signed-rank test (p=0.0078). Moreover, the attention maps of XChondNet explicitly reflect regions consistent with pathologists’ diagnostic concerns, enhancing the interpretability of AI-assisted diagnosis.

## 1. Introduction

Chondrogenic tumors are a heterogeneous group of tumors and a relatively common benign bone tumor. However, there are many difficulties in diagnosis. First, it has a relatively low incidence compared to other bone tumors, such as osteochondroma accounting for 0.44(%) of all bone tumors [1]. As a result, clinicians have relatively little experience in diagnosis, making diagnosis difficult and prone to missed diagnosis or misdiagnosis. Secondly, the complex spatial structural information of the chondrogenic tumors leads to differences in evaluations among doctors. In addition, the limited number of experienced physicians has led to diagnostic delays and misdiagnosis in some cases. However, as technology advances, AI has been increasingly applied to WSI analysis, significantly improving pathological diagnostic performance. It has proven valuable in various tasks, including cancer subtype classification [2,3,4], tumor grading [5,6,7], prognosis analysis [8,9,10], gene mutation prediction [11,12,13] and image generation [14,15,16], improving markedly diagnostic efficiency and accuracy. Thus, AI provides new hope for solving the problem of chondrogenic tumors diagnosis, which can quickly and accurately identify pathological characteristics and provide strong support for doctors. Unlike many tumor classification tasks that mainly rely on local cellular morphology, chondrogenic tumor diagnosis requires the assessment of spatial tissue structures.

**Figure 1:**
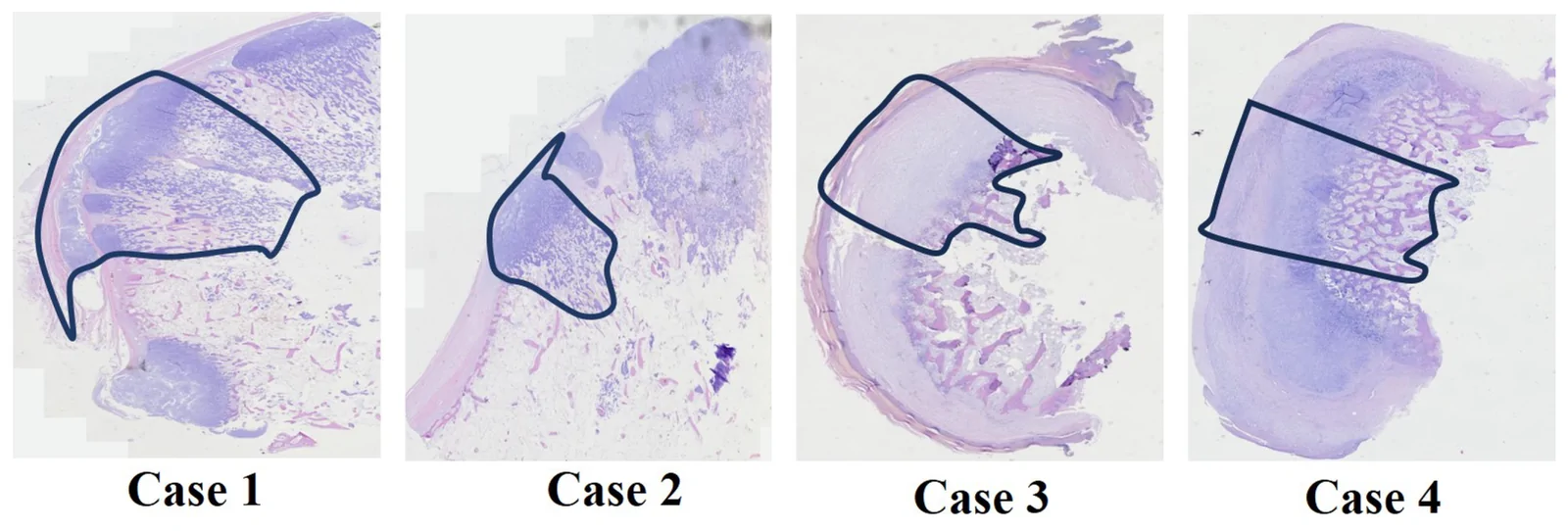
Cases 1 and case 2 are osteochondromas, which are histologically characterized by a typical three-layered spatial structure, fibrous membrane, cartilage and bone. Cases 3 and case 4 are subungual exostoses with histologic features ranging from the peripheral fibrous layer of spindle cells to the germinal active fibroblast layer and then to the cartilage and cartilage osteogenic layer.

Figure 1 shows the lesion areas with spatial structural information for osteochondroma and subungual exostoses in the chondrogenic tumors subtype. Thus, capturing accurate spatial structural information is key to precise chondrogenic tumors diagnosis, but the current methods treat the internal area of the pathological image independently, without fully considering their spatial structural information in the original image and their relationship with each other.

To address this, we propose a XChondNet model for classifying chondrogenic tumors based on WSI, which provides a fusion mechanism of pathological and positional features, as well as a parallel classifier mechanism based on latent encoding. These effectively enable the model to perceive spatial structural information and solve data bias. The main contributions of this study can be summarized as follows:

1. As far as we know, we propose a robust pathological classification model for chondrogenic tumors for the first time.
2. Considering the pathological and positional characteristics of chondrogenic tumors, we propose a mechanism for fusing pathological and positional features, which effectively perceives spatial structural information.
3. Based on the uneven distribution of pathological data of chondrogenic tumors, we propose a parallel classifier mechanism.
4. The focus of XChondNet model is explicitly consistent with that of pathologists, enhancing AI transparency.

The structure of this paper is as follows: Section 2 reviews the related works of pathological diagnosis in chondrogenic tumors and computational pathology; Section 3 introduces the proposed method in detail, including the mechanism for fusing pathological and positional features, as well as the parallel classifier mechanism; Section 4 shows the experimental results and analysis; finally, Section 5 gives the conclusion.

## 2. Related Work

Due to morphological overlap and histological complexity, the diagnosis of enchondromas is challenging. In recent years, computational pathology and deep learning, particularly MIL-based methods, have shown promise in improving diagnostic accuracy. However, limitations remain in spatial structure modeling and data imbalance. This review summarizes relevant research from two perspectives: pathological diagnosis of enchondromas and computational pathology methods.

### 2.1. Pathological Diagnosis of Chondrogenic Tumors

Chondrogenic tumors comprise a heterogeneous group of lesions, including both benign and malignant tumors that typically affect bone tissue. Accurate diagnosis is crucial for developing personalized treatment plans and improving patient prognosis. However, the diagnosis of chondrogenic tumors remains challenging. Jesus-Garcia et al. [17] reported that although PET-CT can reflect metabolic activity, it cannot provide sufficiently clear evidence for distinguishing benign chondroma from chondrosarcoma, indicating the limitations of imaging examinations. Histopathology remains the gold standard for diagnosis, but it is also affected by morphological complexity. Wang et al. [18] discussed the diagnostic difficulty between low-grade chondrosarcoma and enchondroma, as both may show relatively benign chondrocyte morphology without obvious atypia or mitotic activity. Bansal et al. [19] further indicated that chondrogenic tumors need to be distinguished from non-chondrogenic lesions, such as synovial sarcoma, ectopic ossification, and calcified myositis, which may show similar matrix calcification or cell arrangement patterns.

Special histological subtypes also introduce diagnostic traps. Cates et al. [20] and Lan et al. [21] emphasized that chondroblastoma may be misdiagnosed as a malignant tumor because of its cellular polymorphism and nuclear groove characteristics. Schajowicz and Gallardo [22] reported that chondromyxoid fibroma, although benign and rare, often presents heterogeneous histological features such as myxoid matrix and pseudo-lobulated architecture, which may lead to diagnostic confusion. In addition, Bejnordi et al. [23] showed that differences in staining techniques may affect histopathological image analysis, and variability in pathologists’ experience may further influence diagnostic consistency.

With the development of artificial intelligence, deep learning methods have shown great potential in pathological image analysis. Lucas et al. [24] applied deep learning to automatic Gleason pattern classification, while Raees and Thomas [25], Wang et al. [26], and Zhang et al. [27] demonstrated the effectiveness of deep learning in medical image detection and classification tasks. These studies suggest that AI-based methods can provide consistent and efficient diagnostic support, reducing the influence of limited clinical experience and inter-observer variability. Therefore, deep learning has great potential to improve the diagnosis of chondrogenic tumors.

### 2.2. Computational Pathology

In recent years, the proposal and application of deep learning neural networks have greatly improved the performance of AI in medical prediction tasks, and with the continuous advancement of technology, significant improvements have also been made in the diagnosis and treatment of diseases such as cancer. This indicates the significant achievements made in the field of histopathology over the years of development.

In view of the excellent performance of convolutional neural networks (CNNs), many studies have used CNN-based methods or transfer learning techniques for histopathological image classification. De Matos et al. [28] segmented histopathological images into small patches, extracted features using Inception-V3, and combined SVM classifiers for patch screening and classification. Saxena et al. [29] used multiple pretrained CNN models as feature extractors for breast cancer histopathology diagnosis. Nazeri et al. [30] proposed a two-stage CNN framework, where the first stage extracted patch-level features and the second stage learned higher-level image representations for classification.

Due to the extremely high resolution of WSIs, multiple instance learning (MIL) has become a widely used framework for WSI-level classification. Carbonneau et al. [31] provided a comprehensive survey of MIL problem settings and applications. Ilse et al. [32] introduced attention-based deep MIL, enabling the model to learn the contribution of different instances to bag-level prediction. Lu et al. [3] proposed CLAM, which uses attention-based learning and clustering constraints to identify discriminative regions in whole-slide images. Li et al. [33] proposed DSMIL, which jointly models instance-level and bag-level representations through a dual-stream MIL framework. Shao et al. [34] introduced TransMIL by using Transformer-based correlation modeling among instances. Recent studies have also explored medical image super-resolution and attention-guided segmentation methods  [35]. Tang et al. [36] further improved WSI classification through robust feature re-embedding. Xie et al. [37] proposed PHIM-MIL, which constructs hierarchical feature representations for WSI classification. Tang et al.  [38] proposed MHIM-MIL to improve attention-based MIL through masked hard instance mining [39,40,41,42]. These studies demonstrate the effectiveness of deep learning and MIL-based frameworks in computational pathology, but spatial structural information and data imbalance remain insufficiently explored for chondrogenic tumor diagnosis.

In summary, although deep learning models have achieved encouraging results in medical image analysis, especially in tissue pathology image classification, there are still limitations in dealing with the spatial structural information problems. Taking chondrogenic tumors classification as an example, tissue structure characteristics are crucial in diagnosis. For example, the diagnosis of osteochondroma requires attention to obvious pathological manifestations such as the periosteum, cartilage, and mature bone structures. This model also needs to effectively address the challenge of an imbalanced dataset of chondrogenic tumors. However, existing technologies overlook these factors. Based on this, it is urgent to propose a suitable method for the chondrogenic tumors that can perceive spatial structural information and handle data imbalance.

## 3. Method

### 3.1. Notation and Problem Formulation

Given a whole-slide image (WSI), we represent it as a bag of image patches $B={\left\{x_{i}\right\}}_{i=1}^{N}$, where $x_{i}$ denotes the *i*-th patch and *N* is the number of patches in the WSI. The spatial coordinate of $x_{i}$ is denoted as $p_{i}=\left(r_{i},c_{i}\right)\in R^{2}$, where $r_{i}$ and $c_{i}$ are the row and column coordinates, respectively. A pretrained pathological feature extractor maps each patch $x_{i}$ into a pathological feature vector $h_{i}\in R^{d_{h}}$. In this study, pathological features refer to the high-dimensional visual representations extracted from H&E-stained pathological image patches by a pretrained histopathology feature extractor. These features encode local tissue morphology, cellular arrangement, texture patterns, cartilage matrix characteristics, calcification-related structures, and other discriminative visual cues associated with chondrogenic tumor diagnosis. The positional encoding module maps $p_{i}$ into a positional feature vector $e_{i}\in R^{d_{p}}$. The fused patch representation is denoted as $z_{i}\in R^{d}$.

For slide-level classification, each WSI is associated with a slide-level label $y\in {\left\{0,1\right\}}^{K}$, where *K* denotes the number of tumor categories. In this study, K=6, corresponding to OC, ED, CF, SE, CB, and CD. The goal of XChondNet is to learn a slide-level prediction $\hat{y}\in R^{K}$ from the patch-level fused representations ${\left\{z_{i}\right\}}_{i=1}^{N}$.

### 3.2. Overview

**Figure 2:**
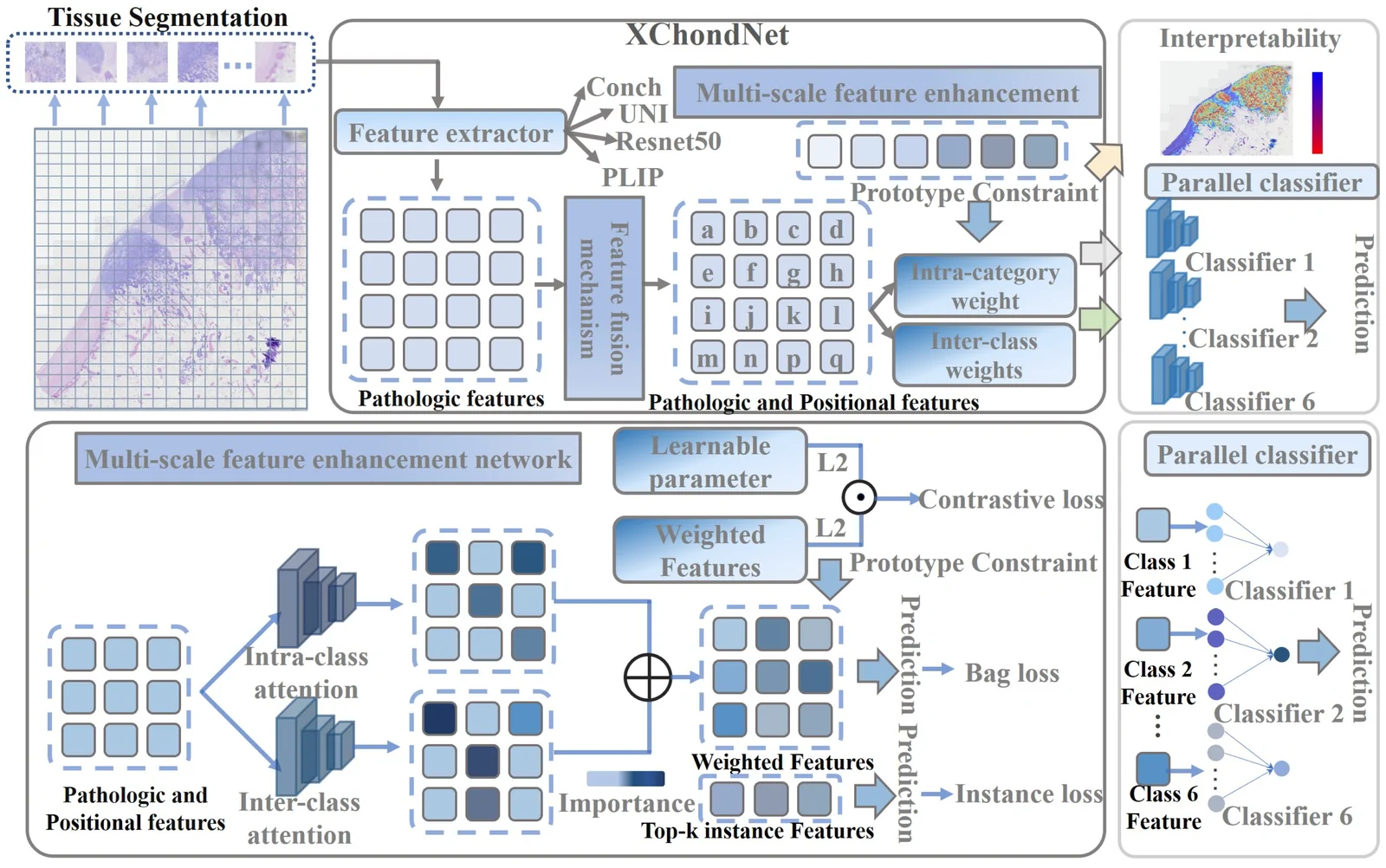
Technical overview of the proposed XChondNet framework for chondrogenic tumor classification. The framework follows a pathology-driven design: tissue regions are first segmented from WSIs, patch-level pathological features and spatial positional features are then extracted and fused to model both local morphology and spatial tissue organization, and the multi-scale feature enhancement network further strengthens discriminative representations through attention modeling, prototype constraints, and contrastive learning. Finally, parallel classifiers are used to improve category-specific discrimination under imbalanced chondrogenic tumor data.

The framework of the proposed model is shown in Figure 2. First, in the data acquisition stage, the tissue samples undergo decalcification, dehydration, transparency, wax immersion, H&E staining, sealing and microscopic observation for about 3 days to form the H&E stained sections we need. Due to the rare quantity of each category in the dataset, we annotated the corresponding regions of interest (ROIs) through the professional pathologist from Tianjin hospital in China and obtained the ROI after processing the whole pathological section. In the data preprocessing stage, the OTSU algorithm is used to separate tissue regions. The positional information are recorded. Then, the pathological features is extracted from the pathological image through the pretrained model, and the position features are formed through the position coding module, and the pathological features is fused with the positional features. Finally, in the prediction stage, the multi-scale feature enhancement network and the parallel classifier mechanism are used to make predictions and generate heatmaps.

The design of XChondNet is motivated by the pathological characteristics of chondrogenic tumors. Unlike many general WSI classification tasks that mainly rely on local cellular morphology, chondrogenic tumor diagnosis requires the assessment of spatial tissue organization, such as the arrangement of fibrous membrane, cartilage, and bone-related regions. Therefore, positional information is incorporated into pathological feature representation to help the model capture spatial structural cues. In addition, because some chondrogenic tumor subtypes are rare and the dataset is imbalanced, a parallel classifier mechanism is designed to enhance category-specific discrimination, see Algorithm 1.

**Algorithm 1** Overall training and inference procedure of XChondNet.**Require:** WSI *X*, slide-level label *y*, pretrained feature extractor ϕ **Ensure:** Slide-level prediction $\hat{y}$ and attention heatmap *H*  1: Segment tissue regions from *X* using OTSU  2: Divide the tissue region into non-overlapping patches
      $B={\left\{\left(x_{i},p_{i}\right)\right\}}_{i=1}^{N}$  3: Extract pathological features
       $h_{i}=\phi \left(x_{i}\right)$  4: Encode spatial coordinates $p_{i}$ into positional features       $e_{i}$  5: Fuse $h_{i}$ and $e_{i}$ to obtain fused representations       $z_{i}$  6: Apply the multi-scale feature enhancement module to model       intra-instance and inter-instance relationships  7: Compute category-aware attention weights and construct       category-specific representations $m_{k}$  8: Compute class-prototype similarities and the prototype-based       contrastive objective  9: Apply the parallel classifiers to obtain category logits       $u_{k}=w_{k}^{⊤}m_{k}+b_{k}$10: Obtain the final slide-level prediction       $\hat{y}=\mathrm{softmax}\left(u\right)$11: During training, optimize the combined objective function12: During inference, map the attention scores of the predicted category       back to the original spatial coordinates13: Generate the attention heatmap *H*14: **return** $\hat{y},H$


### 3.3. Data Preprocessing

After tissue segmentation by the OTSU algorithm, each WSI is divided into non-overlapping patches using a sliding-window strategy. The resulting WSI bag is represented as

(1) $$B={\left\{\left(x_{i},p_{i}\right)\right\}}_{i=1}^{N},$$

where $x_{i}\in R^{s\times s\times 3}$ denotes the *i*-th image patch, $p_{i}=\left(r_{i},c_{i}\right)\in R^{2}$ denotes its spatial coordinate, *s* is the patch size, and *N* is the number of patches in the WSI. This formulation keeps the pathological image patch and its corresponding spatial coordinate explicitly paired, which facilitates subsequent positional feature construction and feature fusion.

### 3.4. Mechanism of Fusion Between Pathological Features and Positional Features

To capture the spatial structural information of chondrogenic tumors, XChondNet integrates pathological features and positional features at the patch level. This module consists of pathological feature extraction, positional feature construction, feature fusion, and multi-scale feature enhancement.

#### 3.4.1. Extracting Pathological Features and Positional Features

For each patch $x_{i}$, the pathological feature represents the morphology-related visual information extracted from the H&E-stained tissue region, including cell distribution, matrix texture, cartilage-related appearance, and ossification- or calcification-related patterns.

For each patch $x_{i}$, a pretrained pathological feature extractor is used to obtain the patch-level pathological feature:

(2) $$h_{i}=\phi \left(x_{i}\right),\;h_{i}\in R^{d_{h}},$$

where ϕ(·) denotes the pretrained pathological feature extractor and $d_{h}$ is the feature dimension.

To encode spatial information, the original coordinate $p_{i}=\left(r_{i},c_{i}\right)$ is first normalized according to the spatial extent of the tissue region:

(3) $${\tilde{r}}_{i}=\frac{r_{i}-\min_{j}\left(r_{j}\right)}{\max_{j}\left(r_{j}\right)-\min_{j}\left(r_{j}\right)+\epsilon },\;{\tilde{c}}_{i}=\frac{c_{i}-\min_{j}\left(c_{j}\right)}{\max_{j}\left(c_{j}\right)-\min_{j}\left(c_{j}\right)+\epsilon },$$

where ϵ is a small constant used to avoid division by zero.

The normalized coordinates are then transformed into high-dimensional positional embeddings through sinusoidal positional encoding. For each dimension *t*, the row-coordinate encoding is defined as

(4) $$PE_{{\tilde{r}}_{i},2t}=\sin\left({\tilde{r}}_{i}/10000^{2t/d_{p}}\right),\;PE_{{\tilde{r}}_{i},2t+1}=\cos\left({\tilde{r}}_{i}/10000^{2t/d_{p}}\right),$$

and the column-coordinate encoding is defined similarly:

(5) $$PE_{{\tilde{c}}_{i},2t}=\sin\left({\tilde{c}}_{i}/10000^{2t/d_{p}}\right),\;PE_{{\tilde{c}}_{i},2t+1}=\cos\left({\tilde{c}}_{i}/10000^{2t/d_{p}}\right).$$

The final positional feature is obtained by concatenating the row and column positional embeddings:

(6) $$e_{i}=\left[PE_{{\tilde{r}}_{i}};PE_{{\tilde{c}}_{i}}\right],$$

where $e_{i}\in R^{d_{p}}$ denotes the positional feature of the *i*-th patch.

#### 3.4.2. Feature Fusion

The pathological feature $h_{i}$ and positional feature $e_{i}$ are concatenated and projected into a unified latent space:

(7) $$z_{i}=W_{f}\left[h_{i};e_{i}\right]+b_{f},\;z_{i}\in R^{d}.$$

The fused patch representations of all patches are denoted as

(8) $$Z={\left[z_{1},z_{2},\ldots ,z_{N}\right]}^{⊤}\in R^{N\times d}.$$

#### 3.4.3. Multi-Scale Feature Enhancement Network

The multi-scale feature enhancement network is designed to strengthen discriminative patch representations and improve slide-level classification. It includes a gated attention module, an instance evaluation module, a region-aware weighting module, and a prototype-based contrastive regularization module.

##### Gated Attention Module

Given the fused feature matrix $Z\in R^{N\times d}$, a gated attention mechanism is used to estimate category-aware patch importance:

(9) $$A=\tanh\left(ZW_{a}+b_{a}\right),\;G=\sigma \left(ZW_{g}+b_{g}\right),$$

(10) $$\alpha ={\mathrm{softmax}}_{N}\left(\left(A⊙G\right)W_{\alpha }\right),$$

where A and G denote the nonlinear feature branch and the gating branch, respectively, ⊙ denotes element-wise multiplication, and $\alpha \in R^{N\times K}$ denotes the category-aware attention weights. The softmax operation is performed over the patch dimension for each class.

##### Instance Evaluation Module

To enhance instance-level discrimination, we select high-attention and low-attention patches according to the category-aware attention weights. For the ground-truth class $k^{*}$, the positive and negative instance sets are defined as

(11) $$S_{pos}=\mathrm{TopK}\left({\left\{\alpha_{i,k^{*}}\right\}}_{i=1}^{N},K_{\mathrm{ins}}\right),\;S_{neg}=\mathrm{TopK}\left({\left\{-\alpha_{i,k^{*}}\right\}}_{i=1}^{N},K_{\mathrm{ins}}\right),$$

where $K_{\mathrm{ins}}$ is the number of selected instances. The selected instances are then used for instance-level supervision.

##### Region-Aware Feature Weighting Module

A region-aware weight $\beta \in R^{N}$ is further generated to describe the importance of each patch in the feature space:

(12) $$\beta ={\mathrm{softmax}}_{N}\left(W_{\beta 2}\mathrm{ReLU}\left(ZW_{\beta 1}+b_{\beta 1}\right)+b_{\beta 2}\right).$$

The final slide-level representation for each category is obtained by combining category-aware attention and region-aware weights:

(13) $$m_{k}=\sum_{i=1}^{N}\alpha_{i,k}\beta_{i}z_{i},\;k=1,\ldots ,K.$$

The category-specific slide representations are denoted as

(14) $$M={\left[m_{1},m_{2},\ldots ,m_{K}\right]}^{⊤}\in R^{K\times d}.$$

##### Prototype-Based Contrastive Regularization Module

For prototype-based feature regularization, we define a learnable prototype matrix $C\in R^{K\times d}$, where each row corresponds to a class prototype. The category-specific representations are first aggregated into a global slide representation:

(15) $$\bar{m}=\frac{1}{K}\sum_{k=1}^{K}m_{k}.$$

The similarity between the slide-level representation and class prototypes is computed as

(16) $$o^{proto}=\frac{\mathrm{Norm}\left(\bar{m}\right)\mathrm{Norm}{\left(C\right)}^{⊤}}{\tau },\;o^{proto}\in R^{K},$$

where Norm(·) denotes L2 normalization, τ is the temperature parameter, and $o^{proto}$ denotes the prototype similarity logits.

### 3.5. The Parallel Classifier Mechanism

Due to the rarity of chondrogenic tumors, the dataset shows obvious category imbalance. To reduce mutual interference among different categories, XChondNet adopts a parallel classifier mechanism, where each category is assigned an independent classifier. This design encourages each classifier to focus on category-specific discriminative features.

Given the category-specific representation $m_{k}$, the logit of the *k*-th class is computed as

(17) $$u_{k}=w_{k}^{⊤}m_{k}+b_{k},\;k=1,\ldots ,K,$$

where $w_{k}$ and $b_{k}$ are the parameters of the *k*-th classifier. The logits of all categories are collected as $u=[u_{1},u_{2},\ldots ,u_{K}]$. The final slide-level probability is obtained by

(18) $$\hat{y}=\mathrm{softmax}\left(u\right).$$

### 3.6. Attention-Based Interpretability Analysis

The interpretability of XChondNet is derived from its intrinsic category-aware attention mechanism. Specifically, patch-level attention scores are used to estimate the relative contribution of different tissue regions to the slide-level prediction, and these scores are mapped back to their original spatial positions to generate attention heatmaps.

Given the fused patch representation matrix $Z\in R^{N\times d}$, XChondNet first calculates the gated attention representation:

(19) $$U=\tanh\left(ZW_{a}+b_{a}\right)⊙\sigma \left(ZW_{g}+b_{g}\right),$$

where ⊙ denotes element-wise multiplication, and σ(·) denotes the sigmoid activation function. The category-aware attention weights are then obtained by

(20) $$\alpha ={\mathrm{softmax}}_{N}\left(UW_{\alpha }\right),$$

where $\alpha \in R^{N\times K}$ denotes the attention weight matrix, *N* is the number of patches, and *K* is the number of tumor categories. The softmax operation is performed along the patch dimension for each category, so that the attention weights reflect the relative importance of different patches for a specific class.

For the predicted class $\hat{k}$, the attention score of the *i*-th patch is defined as

(21) $$s_{i}=\alpha_{i,\hat{k}}.$$

To generate the heatmap, the attention scores are normalized to the range of 0 to 1:

(22) $${\tilde{s}}_{i}=\frac{s_{i}-\min_{j}\left(s_{j}\right)}{\max_{j}\left(s_{j}\right)-\min_{j}\left(s_{j}\right)+\epsilon },$$

where ϵ is a small constant used to avoid division by zero. The normalized attention score ${\tilde{s}}_{i}$ is then assigned to the original spatial coordinate $p_{i}=\left(r_{i},c_{i}\right)$ of the corresponding patch:

(23) $$H\left(p_{i}\right)={\tilde{s}}_{i}.$$

Finally, the attention map H is converted into a color heatmap and overlaid on the original WSI. Regions with higher attention scores indicate tissue regions that contribute more strongly to the slide-level prediction, thereby providing visual evidence for the diagnostic decision of XChondNet.

### 3.7. Loss Function

The overall training objective consists of the slide-level classification loss, the instance-level loss, and the prototype contrastive loss:

(24) $$L_{total}=L_{bag}+L_{inst}+L_{proto}.$$

The slide-level classification loss is defined as

(25) $$L_{bag}=-\sum_{k=1}^{K}y_{k}\log\left({\hat{y}}_{k}\right),$$

where $y=[y_{1},\ldots ,y_{K}]$ is the one-hot slide-level label, and $\hat{y}=\left[{\hat{y}}_{1},\ldots ,{\hat{y}}_{K}\right]$ is the predicted probability distribution.

For instance-level supervision, the selected positive and negative instances are optimized using binary cross-entropy:

(26) $$L_{inst}=-\frac{1}{\left|S\right|}\sum_{i\in S}\left[q_{i}\log\left({\hat{q}}_{i}\right)+\left(1-q_{i}\right)\log\left(1-{\hat{q}}_{i}\right)\right],$$

where $S=S_{pos}\cup S_{neg}$ denotes the set of selected instances, $q_{i}$ is the pseudo-label of the selected instance, and ${\hat{q}}_{i}$ is the predicted probability of the instance classifier.

The prototype contrastive loss is defined as

(27) $$L_{proto}=-\sum_{k=1}^{K}y_{k}\log\frac{\exp(o_{k}^{proto})}{\sum_{j=1}^{K}\exp\left(o_{j}^{proto}\right)},$$

where $o_{k}^{proto}$ denotes the prototype similarity logit for the *k*-th class.

## 4. Experiments

### 4.1. Data Acquisition

**Figure 3:**
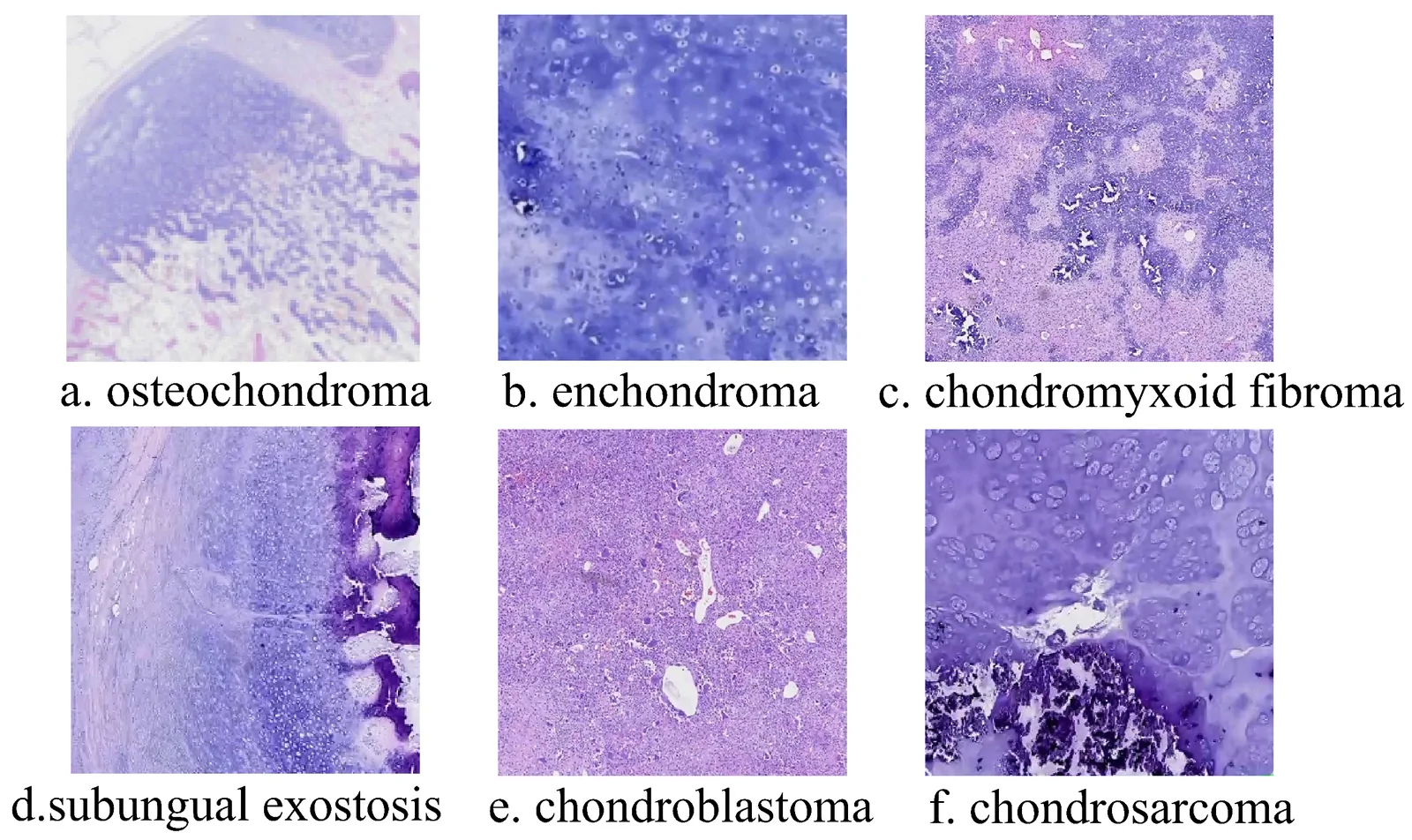
Representative histopathological images of six categories of chondrogenic tumors: (**a**) osteochondroma, (**b**) enchondroma, (**c**) chondromyxoid fibroma, (**d**) subungual exostosis, (**e**) chondroblastoma, and (**f**) chondrosarcoma.

In this study, we used the H&E-stained chondrogenic tumor dataset provided by Tianjin Hospital, China, which consists of uncompressed whole-slide images (WSIs). The dataset was derived from archived pathological specimens of 228 patients with chondrogenic tumors collected between January 2017 and September 2024. Since multiple pathological sections may be available for a single patient, the original cohort contained 264 pathological samples in total. The images were scanned at 20× magnification with a pixel resolution of 0.2μm/pixel, and the dimensions of the WSIs varied across samples. During the quality-control process, sections with poor staining quality or severe image degradation were excluded. In particular, some older archived sections exhibited staining deterioration associated with prolonged storage, which affected the visibility of pathological structures and could interfere with reliable pathological interpretation. Therefore, these sections were removed during the screening process. After quality screening, 139 samples were retained for subsequent analysis. We acknowledge that this quality-control procedure may introduce potential selection bias, as the retained samples may have relatively better staining quality and clearer tissue morphology than the excluded sections. Consequently, the generalizability of XChondNet to older slides, slides with substantial staining variation, or slides containing severe artifacts may be limited, see Figure 3.

In view of the limited number of samples in the dataset, we annotated the ROI in the WSIs by professional pathologists in Tianjin hospital, and extracted 1472 ROIs from them for experiments. To deal with the imbalance of categories in the dataset, and ensure the comprehensiveness of model training and the reliability of evaluation results, we used the 5-fold cross validation method to divide the dataset into the training set, the validation set and the test set. The ratio of each patient level was set as 8:1:1. In this process, we ensured that all samples of the same patient were assigned to the same subset. Taking the first fold cross validation as an example, we allocated 1147 ROIs for the training set, 162 ROIs for the validation set, and 163 ROIs for the test set. The specific division of the dataset is shown in Table 1.

### 4.2. Experimental Details

During training, XChondNet was optimized using the Adam optimizer with an initial learning rate of 0.001 and a weight decay of $1\times 10^{-5}$. The size of the small batch is 1. The classifier will verify the dataset every 200 epochs trained, and apply the early stop mechanism to finally test the test set. ACC, AUC, F1 score, Recall and Precision were used as experimental evaluation indicators. All experiments were performed on two NVIDIA RTX 3090 GPU computers.

### 4.3. Experimental Evaluation Indicators

This paper uses ACC, AUC, F1 score, Recall and Precision as evaluation indicators.

Accurate prediction of proportion image is called accuracy. It is indicated as follows:

(28) $$\mathrm{Accuracy}=\frac{TP+TN}{TP+TN+FP+FN}$$

The accuracy rate is measured by calculating the proportion of correct judgment of the model in all predictions, in which the true case (*TP*), the true negative case (*TN*), the false positive case (*FP*) and the false negative case (*FN*) are expressed by their abbreviations respectively.

AUC is an indicator calculated based on receiver operating characteristic curve. The ROC curve is drawn with the false positive rate (FPR) as the horizontal axis and the true positive rate (TPR) as the vertical axis. The value of AUC is between 0 and 1, indicating the ability of the model to distinguish between positive and negative samples at different thresholds. The closer the AUC is to 1, the better the performance of the model.

Recall rate refers to the proportion of the number of samples correctly predicted as positive by the model to the total number of actual positive samples.

(29) $$\mathrm{Recall}=\frac{TP}{TP+FN}$$

Precision measures how accurate a model’s positive class predictions are.

(30) $$Precision=\frac{TP}{FP+TP}$$

This indicator can evaluate the overall validity value of the model.

(31) $$F1\;\mathrm{Score}=2\times \frac{\mathrm{precision}\times \mathrm{recall}}{\mathrm{precision}+\mathrm{recall}}$$

### 4.4. Comparative Experiment

To validate the effectiveness of the XChondNet model, we extracted features using four different feature extractors (Conch, ResNet-50, PLIP and UNI). We then compared our approach with thirteen representative techniques: MIL, Max-MIL, Mean-MIL, Att-MIL, Gatt-MIL, CLAM-SB, CLAM-MB, AB-MIL, MHIM-MIL, TRANS-MIL, DS-MIL, DTFD-MIL, and RRT-MIL. All methods were implemented based on their official code and evaluated using a unified experimental setup. The average performance metrics were obtained using 5-fold cross-validation.

To make the comparison setting clearer, we briefly describe the compared MIL-based methods. MIL, Max-MIL, and Mean-MIL are conventional MIL baselines that aggregate instance-level information using standard pooling strategies. Att-MIL and AB-MIL introduce attention-based aggregation mechanisms to learn the contribution of different instances to bag-level prediction. Gatt-MIL further adopts a gated attention mechanism to enhance instance weighting. CLAM-SB and CLAM-MB introduce clustering-constrained attention learning for single-branch and multi-branch MIL classification, respectively. DSMIL models instance-level and bag-level representations jointly through a dual-stream design. TRANS-MIL introduces Transformer-based modeling to capture correlations among instances. MHIM-MIL improves attention-based MIL by masked hard instance mining, while DTFD-MIL and RRT-MIL are recent strong MIL methods designed to improve feature distillation and robust representation learning. These methods cover conventional pooling-based MIL, attention-based MIL, Transformer-based MIL, and recent robust MIL frameworks, providing a comprehensive comparison for evaluating XChondNet.

Table 2 summarizes the comparative performance of different MIL-based methods across four feature extractors. Conventional pooling-based approaches, such as Max-MIL, and Mean-MIL, exhibit relatively low accuracy and unstable Recall, reflecting their limited ability to capture the complex structural information of chondrogenic tumors pathology. Advanced methods, including CLAM, TRANS-MIL, and DSMIL, achieve moderate improvements, but their performance remains inconsistent across feature extractors. In contrast, the proposed XChondNet consistently achieves the best or near best results in all metrics, with particularly notable gains in AUC and Recall. This improvement validates the effectiveness of our pathological and positional feature fusion mechanism in perceiving spatial structural cues, while the parallel classifier strategy enhances robustness against data imbalance.

**Figure 4:**
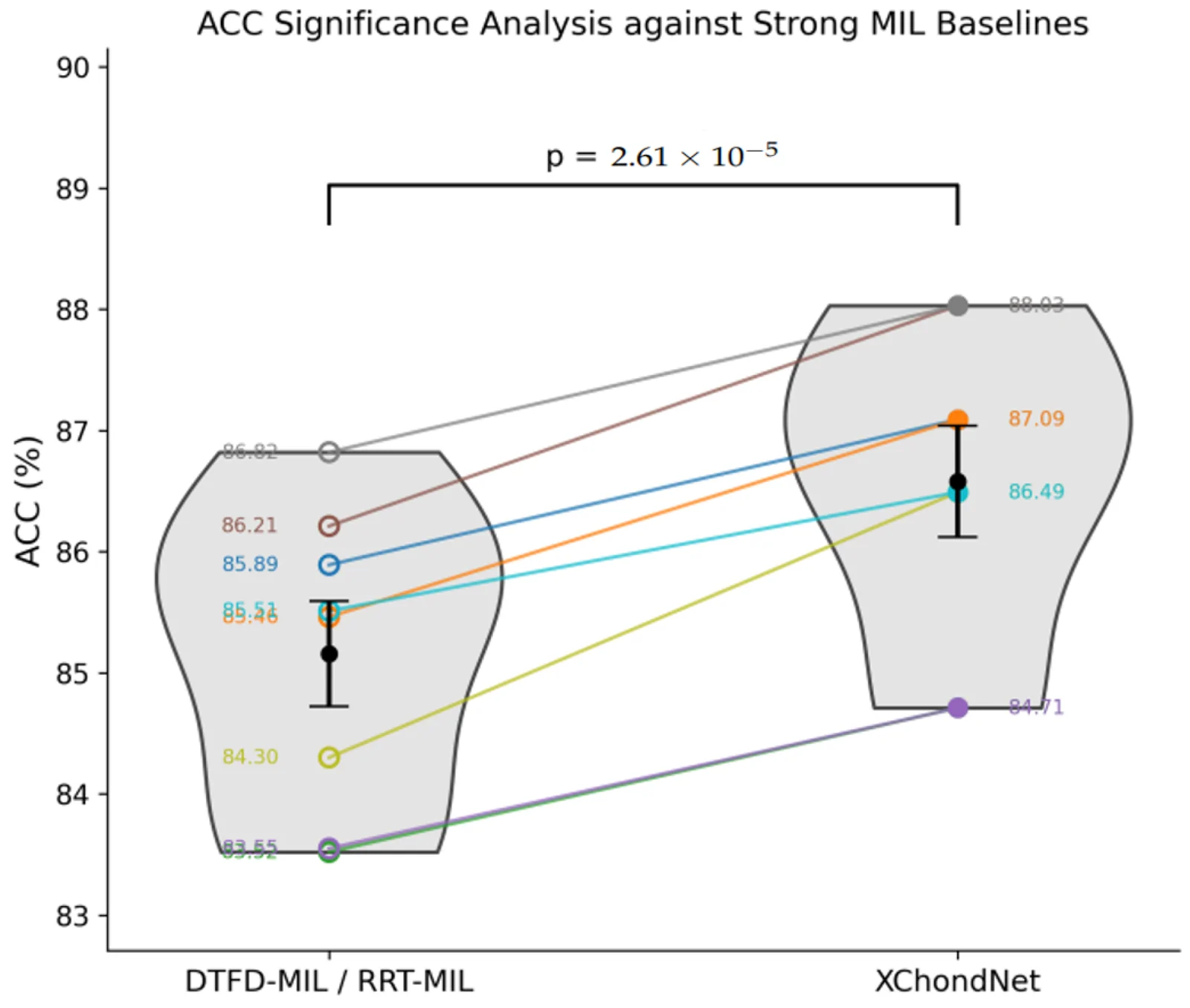
Paired statistical significance analysis of ACC between XChondNet and two strong MIL baselines, DTFD-MIL and RRT-MIL. The comparison was conducted across four feature extractors, including Conch, ResNet-50, UNI, and PLIP.

To further validate the statistical reliability of the comparative experiments, we conducted paired significance analysis on ACC between XChondNet and two strong MIL baselines, DTFD-MIL and RRT-MIL. These two methods were selected because they achieved the best or near-best performance among the compared MIL-based methods in Table 2. Specifically, the comparison was conducted across four feature extractors, including Conch, ResNet-50, UNI, and PLIP, resulting in eight paired comparisons. As shown in Figure 4, XChondNet consistently outperforms DTFD-MIL and RRT-MIL under different feature extractor settings. The average ACC of the two strong baselines is 85.16%, whereas XChondNet achieves an average ACC of 86.58%, corresponding to an average improvement of 1.42%. The paired significance analysis further confirms that this improvement is statistically significant, with a paired *t*-test result of $p=2.61\times 10^{-5}$ and a Wilcoxon signed-rank test result of p=0.0078. These results indicate that the superiority of XChondNet is not only reflected in higher average performance, but is also statistically supported when compared with competitive MIL methods from the literature.

**Figure 5:**
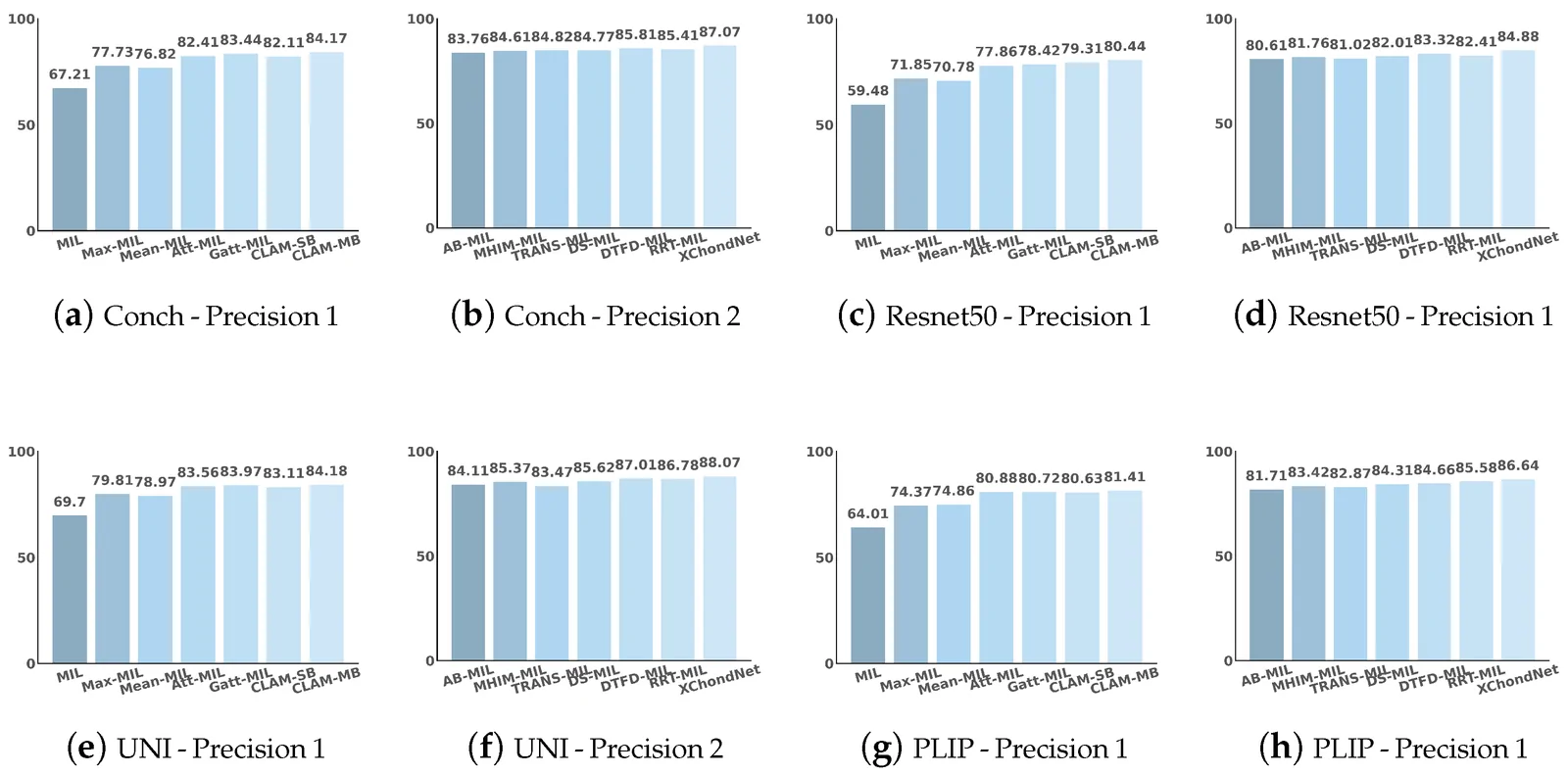
Visualization of the Precision performance in comparative experiments across the Conch, Resnet50, UNI and PLIP feature extractor.

The Precision visualizations in Figure 5 further confirm these findings. XChondNet maintains stable and superior accuracy. This stability indicates that the model’s attention aligns with clinically relevant regions. Taken together, these results demonstrate XChondNet’s consistent advantages over existing MIL frameworks, supporting its potential for practical application in pathology diagnosis.

### 4.5. Why We Choose the Mechanism of Fusing Pathological and Positional Features?

To further validate the contribution of each component, we conducted a set of ablation experiments. Here, w/o denotes “without.” Specifically, w/o position features removes the positional encoding branch and relies solely on pathological features; w/o intra-class attention discards the intra-class attention module; w/o inter-class attention eliminates the inter-class attention mechanism to break structural dependencies across classes; w/o prototype constraint drops the prototype-based regularization loss while keeping the rest unchanged.

For clarity, the following abbreviations are used in the ablation study: w/o pos denotes the removal of position features, i.e., the model relies solely on pathological features; w/o Intra indicates the elimination of the intra-class attention module; w/o Inter refers to the removal of the inter-class attention mechanism, thereby breaking structural dependencies across classes; and w/o Pro represents the exclusion of the prototype-based regularization constraint while keeping other components unchanged.

**Figure 6:**
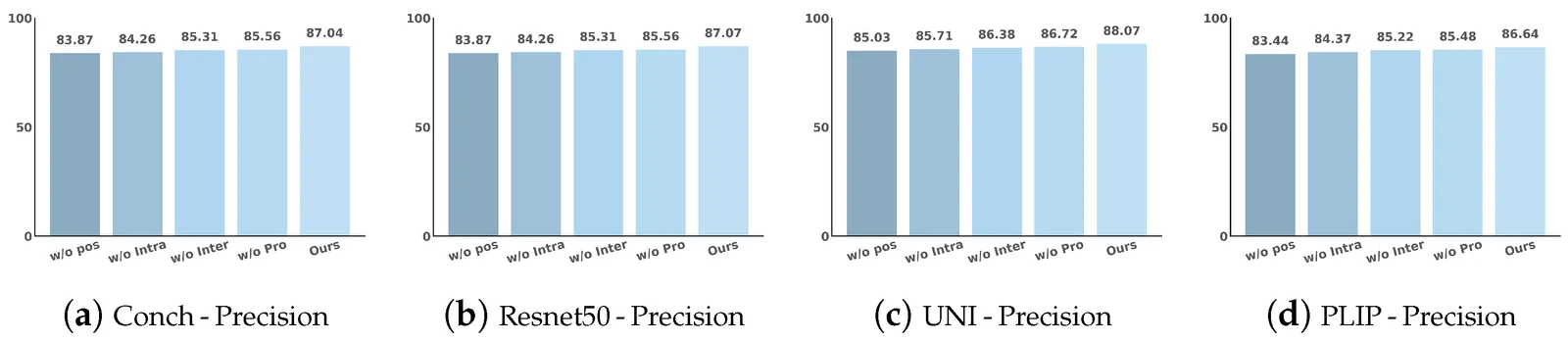
Visualization of the Precision performance in ablation experiments across the Conch, Resnet50, UNI and PLIP feature extractor.

Table 3 summarizes the ablation study results, while Figure 6 further illustrates the Precision evaluation metric across different settings. The complete model achieves the best performance in terms of ACC, AUC, F1, and Recall, confirming the effectiveness of the proposed design. Among the components, w/o pos leads to the most significant degradation, highlighting the critical role of spatial information in pathological representation of Chondrogenic tumors. w/o Intra demonstrates the necessity of modeling local dependencies among instances. In contrast, w/o Inter and w/o Pro causes relatively smaller reductions, but both still contribute to improving global structure modeling.

### 4.6. Why We Choose the Parallel Classifier Mechanism?

To assess the contribution of the parallel classifier mechanism, we performed an ablation study using features extracted from four different feature extractors. The full model was compared with variants w/o PC (without the parallel classifier), where each feature set was processed by a single classifier instead of parallel classifiers, while all other components remained unchanged. In the results table, **✓** indicates the presence of the parallel classifier mechanism, and **✗** indicates its absence.

**Figure 7:**
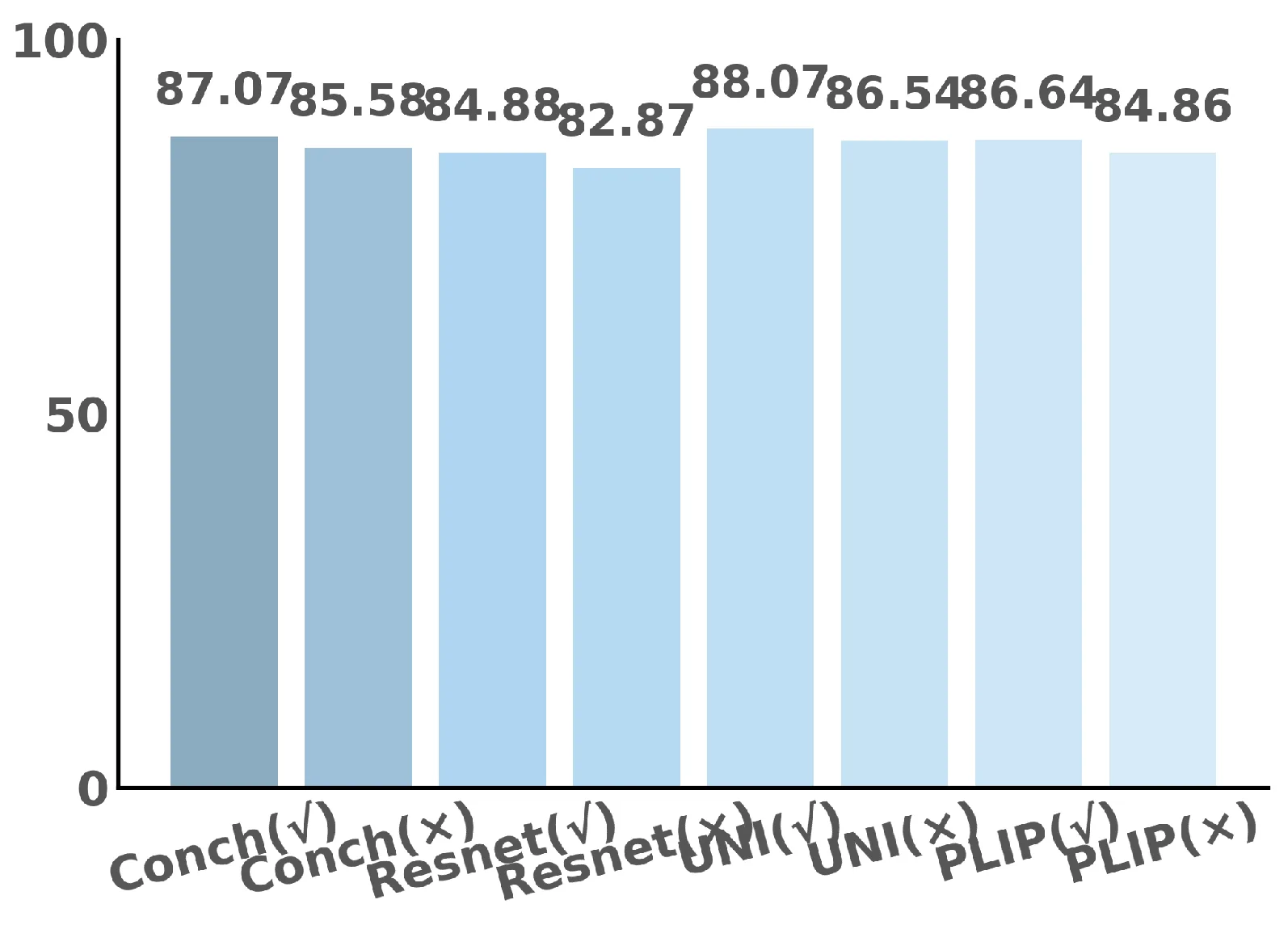
Ablation experiment precision of the parallel classifier mechanism.

The ablation results across the four feature extractors consistently demonstrate the effectiveness of the parallel classifier mechanism. As shown in Table 4 and Figure 7, removing this mechanism leads to a degradation across all evaluation metrics. In contrast, the full model with **✓** achieves consistently higher Precision, Recall, and AUC, indicating that parallel classifiers can better exploit the complementary information from multiple feature representations. These findings confirm that the parallel classifier mechanism enhances the discriminative capability of the proposed framework.

**Figure 8:**
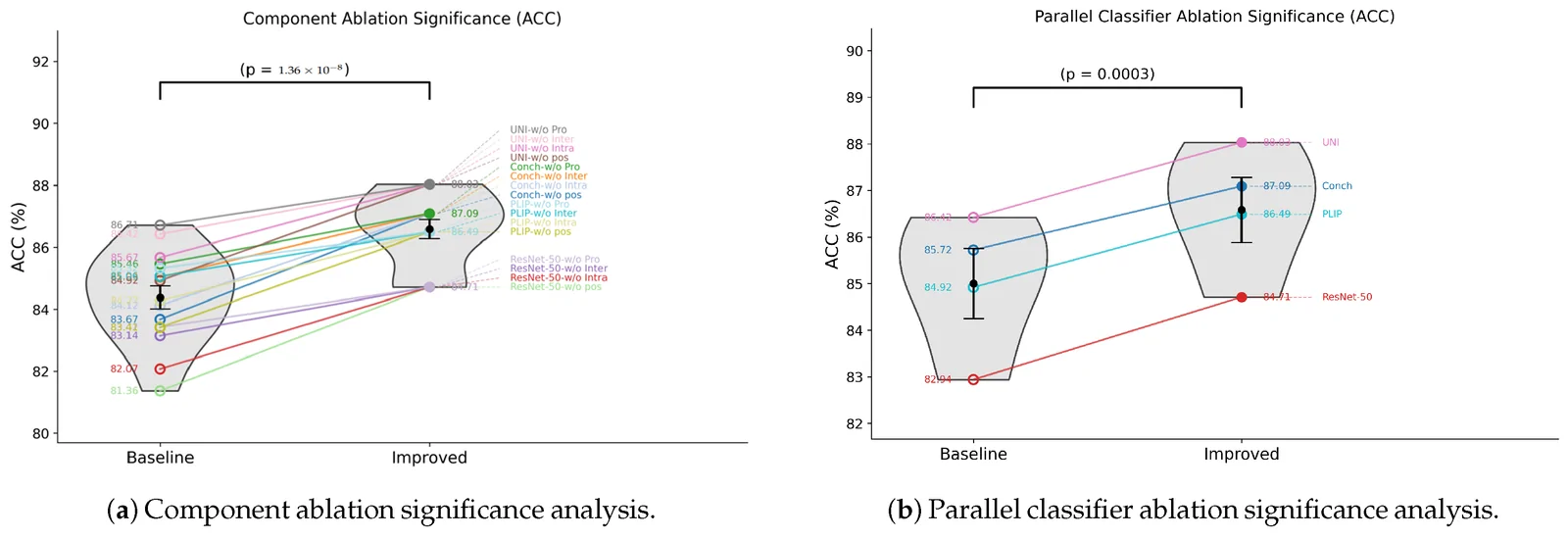
Paired statistical significance analysis of the ablation experiments on ACC. The full XChondNet shows significant improvements over the ablated variants in the component ablation study, and the parallel classifier mechanism also brings statistically significant performance gains across different feature extractors.

To further validate the reliability of the ablation results, we conducted paired statistical significance analysis on the ablation experiments. For the component ablation study, the full XChondNet was compared with its ablated variants under the same feature extractor settings, including the variants without positional features, intra-class attention, inter-class attention, and prototype constraint. As shown in Figure 8, XChondNet consistently achieved higher ACC values than the corresponding ablated variants across different feature extractors, and the improvement was statistically significant with a paired test result of $p=1.36\times 10^{-8}$. This indicates that the proposed positional modeling, intra-class attention, inter-class attention, and prototype constraint jointly contribute to more effective pathological feature representation.

For the parallel classifier ablation, we further compared XChondNet with and without the parallel classifier mechanism. The paired analysis across different feature extractors showed that introducing the parallel classifier led to consistent performance gains, with a statistically significant improvement of p=0.0003. This result demonstrates that the parallel classifier can enhance category-specific discrimination, which is particularly beneficial for chondrogenic tumor diagnosis with imbalanced subtype distributions.

### 4.7. Temporal Validation and Domain-Shift Analysis

To further evaluate the generalization ability of XChondNet and reduce the potential risk of overfitting caused by the limited dataset size, we conducted additional temporal validation and domain-shift robustness analyses. For temporal validation, all patients were split according to the chronological order of sample collection. Cases collected from 2017 to 2022 were used for training and validation, while cases collected from 2023 to 2024 were held out as an independent temporal test set. This setting is more challenging than random cross-validation because the test samples are temporally separated from the training data, thereby providing a more realistic evaluation of model robustness over time. Importantly, the temporal split was performed at the patient level to avoid information leakage.

As shown in Table 5, XChondNet maintained stable diagnostic performance on the temporally independent test set. Under the same UNI feature extractor and temporal split setting, XChondNet achieved an ACC of 86.82%, AUC of 97.23%, F1 score of 86.87%, and Recall of 86.76%. Compared with two strong MIL baselines, XChondNet outperformed DTFD-MIL by 1.35% in ACC and RRT-MIL by 0.89% in ACC. Similar improvements were also observed in F1 score and Recall. These results suggest that XChondNet can maintain relatively robust diagnostic performance on chronologically separated cases, indicating its potential temporal generalization ability.

In addition, we performed a domain-shift robustness analysis to evaluate whether XChondNet remains stable under different feature-domain settings. Specifically, different pathological feature extractors, including Conch, ResNet-50, UNI, and PLIP, were regarded as different feature-domain settings because they generate different representation spaces. To make the analysis more convincing, XChondNet was compared with two strong MIL baselines, DTFD-MIL and RRT-MIL, under the same feature-domain settings.

As shown in Table 6, XChondNet consistently achieved higher ACC than DTFD-MIL and RRT-MIL across all four feature-domain settings. Specifically, XChondNet obtained a mean ACC of 86.58%, outperforming DTFD-MIL and RRT-MIL, which achieved mean ACC values of 84.98% and 85.34%, respectively. The average ACC improvements over DTFD-MIL and RRT-MIL were 1.60% and 1.24%, respectively. These results indicate that the proposed framework is not overly dependent on a single feature extractor and can maintain robust diagnostic performance under different feature-domain conditions.

### 4.8. Statistical Analysis and Reliability Evaluation

To further support the reliability of the final comparison, we added a comprehensive statistical analysis, including confidence interval estimation, paired significance testing, bootstrap analysis, ROC confidence-band visualization, and per-class performance evaluation. Since DTFD-MIL and RRT-MIL achieved strong performance among the compared MIL methods, they were selected as representative baselines for comparison with XChondNet.

The confidence interval results in Table 7 show that XChondNet achieved the highest ACC, F1 score, and Recall among the compared methods. Although RRT-MIL obtained a slightly higher AUC, XChondNet showed better overall diagnostic performance with stable confidence intervals, supporting the reliability of the final comparison.

We further performed paired statistical significance tests to evaluate whether the ACC improvements of XChondNet over strong MIL baselines were statistically reliable. Specifically, Conch, ResNet-50, UNI, and PLIP were regarded as four paired feature-domain settings. XChondNet was compared with DTFD-MIL and RRT-MIL under the same feature extractor settings.

The significance analysis in Table 8 shows that XChondNet achieved consistent ACC improvements over DTFD-MIL and RRT-MIL across different feature-domain settings. Compared with DTFD-MIL and RRT-MIL, XChondNet improved the mean ACC by 1.60% and 1.25%, respectively. The paired *t*-test showed statistically significant improvements for both comparisons. When the two strong baselines were jointly considered, XChondNet achieved an average ACC improvement of 1.42%, with significant results in both the paired *t*-test ($p=2.61\times 10^{-5}$) and the Wilcoxon signed-rank test (p=0.0078). These results indicate that the marginal ACC gains of XChondNet are statistically supported across different feature-domain settings.

To further estimate the uncertainty of the final performance, we performed bootstrap analysis for XChondNet under the UNI feature extractor. The test samples were repeatedly resampled with replacement, and the evaluation metrics were recalculated for each bootstrap sample. The 2.5th and 97.5th percentiles of the bootstrap distribution were used as the 95% confidence interval.

Table 9 shows that XChondNet achieved stable bootstrap confidence intervals across the main evaluation metrics. The ACC, F1 score, and Recall confidence intervals were centered around the observed values, while the AUC confidence interval remained at a high level. These results indicate that the final performance estimation of XChondNet is stable under bootstrap resampling.

**Figure 9:**
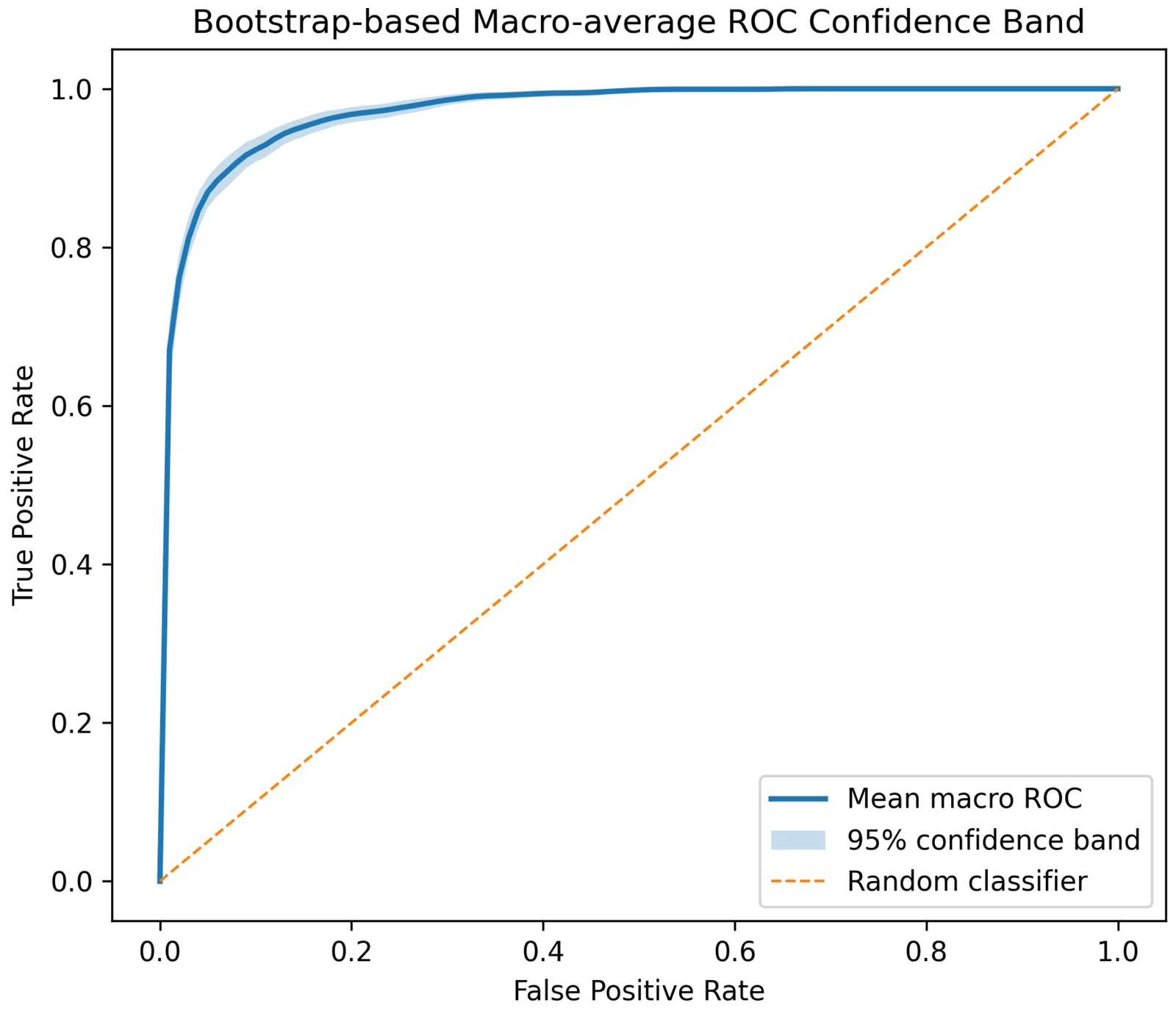
Bootstrap-based macro-average ROC confidence band of XChondNet under the UNI feature extractor. The solid curve represents the mean macro-average ROC curve, and the shaded region indicates the 95% confidence band estimated by bootstrap resampling.

In addition, we generated a bootstrap-based macro-average ROC confidence band to visualize the stability of the ROC curve under resampling. Figure 9 shows the ROC curve remained close to the upper-left region, and the confidence band was relatively narrow, indicating stable discrimination performance of XChondNet under bootstrap resampling.

As is shown Table 10, we further conducted a per-class performance analysis to evaluate the diagnostic ability of XChondNet for each subtype of chondrogenic tumors. Precision, Recall, F1 score, and AUC were calculated for each class under the UNI feature extractor.

**Figure 10:**
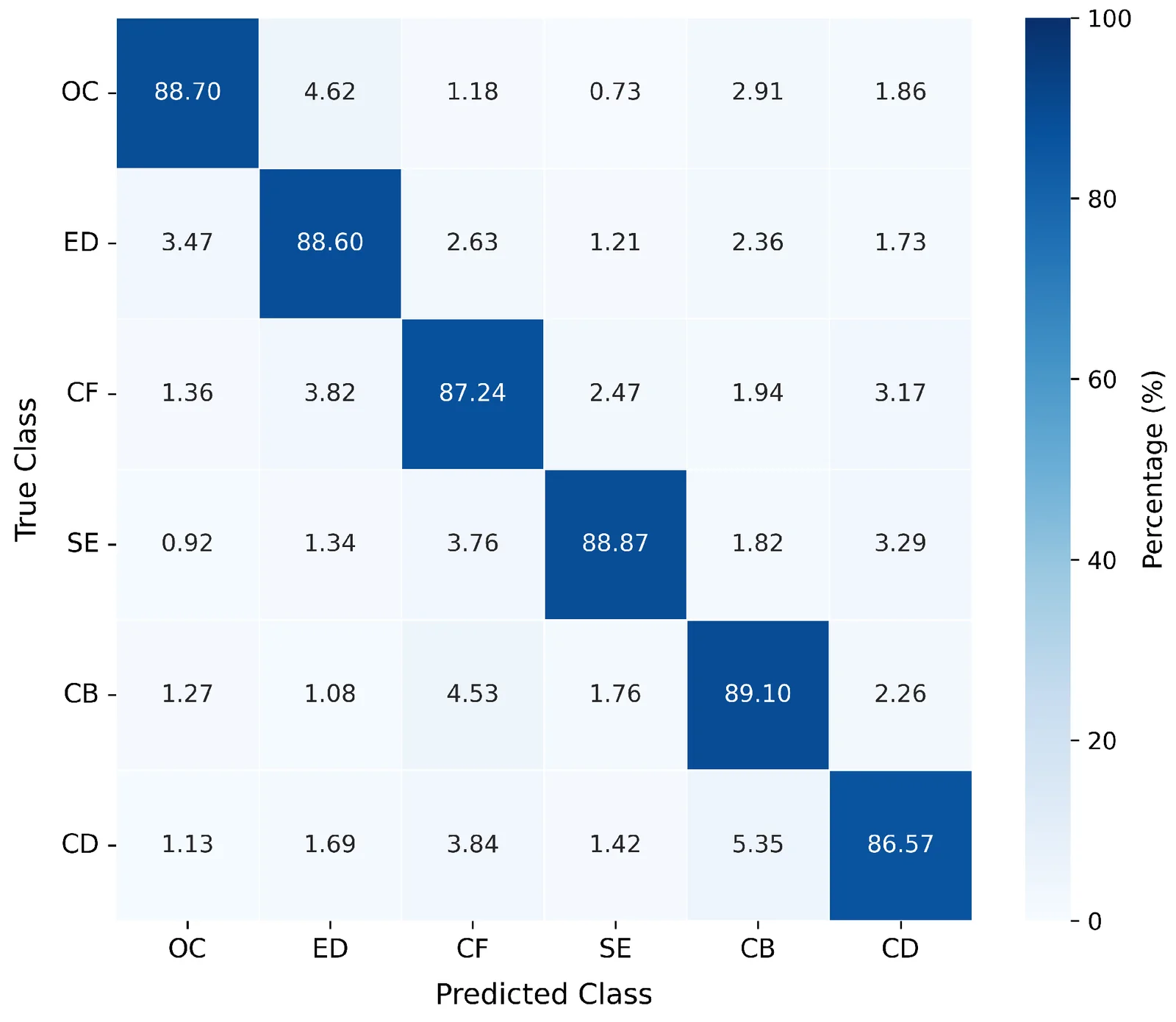
Normalized confusion matrix of XChondNet on the held-out test set of Fold 1 using the UNI feature extractor. The rows represent the true classes and the columns represent the predicted classes. Values are reported as percentages.

To further investigate the class-specific prediction patterns of XChondNet, we analyzed the normalized confusion matrix on the held-out test set of Fold 1 using the UNI feature extractor, as shown in Figure 10. Overall, XChondNet achieved high recognition rates across all six classes, with the diagonal entries ranging from 86.57% to 89.10%. This indicates that the model can effectively distinguish the major chondrogenic tumor subtypes.

Nevertheless, some class-specific misclassification patterns remain. In particular, chondrosarcoma (CD) achieved a Recall of 86.57%, with 5.35% of CD cases misclassified as CB and 3.84% misclassified as CF. Conversely, a proportion of CF and CB cases were also predicted as CD, suggesting bidirectional confusion between chondrosarcoma and these subtypes. Such confusion may be attributed to the substantial morphological overlap among cartilaginous tumors, where differences in cellularity, matrix composition, and architectural patterns can be subtle and heterogeneous across tissue regions. Other classes showed relatively dispersed misclassification patterns, with no single competing class accounting for a dominant proportion of errors.

As is shown 10, these observations suggest that, although XChondNet provides robust overall discrimination, distinguishing chondrosarcoma from morphologically similar cartilaginous tumor subtypes remains challenging. The confusion matrix therefore provides complementary evidence to the class-level Precision, Recall, and F1 score results and helps identify specific areas where further improvement may be beneficial.

### 4.9. Interpreting Model Predictions Through Attention Heatmaps

To intuitively explain the relative importance assigned by XChondNet to different tissue regions, we first calculated the non-normalized attention scores of all extracted patches in each slide. These scores were then converted into percentile scores and normalized to the range of 0 to 1. Next, the normalized attention scores were mapped to RGB colors using a diverging color map, where high-attention regions are represented in red and low-attention regions are represented in blue. Finally, the generated heatmap was superimposed on the original WSI with a transparency of 0.5, allowing the model-highlighted regions and the underlying morphological structures of the H&E-stained slide to be visualized simultaneously.

Due to the limited number of available pathological samples, pathologist-annotated ROIs were used as the basic analysis units for model training and evaluation. Importantly, the test set was strictly held out from model training and was not used during model optimization. For interpretability analysis, we used the held-out test samples and their corresponding complete WSIs. Specifically, the attention scores generated for the patches within the test ROIs were mapped back to their original spatial coordinates in the corresponding complete WSIs. Therefore, although the ROIs were used for model training and evaluation, the attention heatmaps were visualized on the complete WSIs rather than being restricted to the manually cropped ROI regions. This enables the spatial distribution of model attention to be examined in the context of the entire pathological slide.

**Figure 11:**
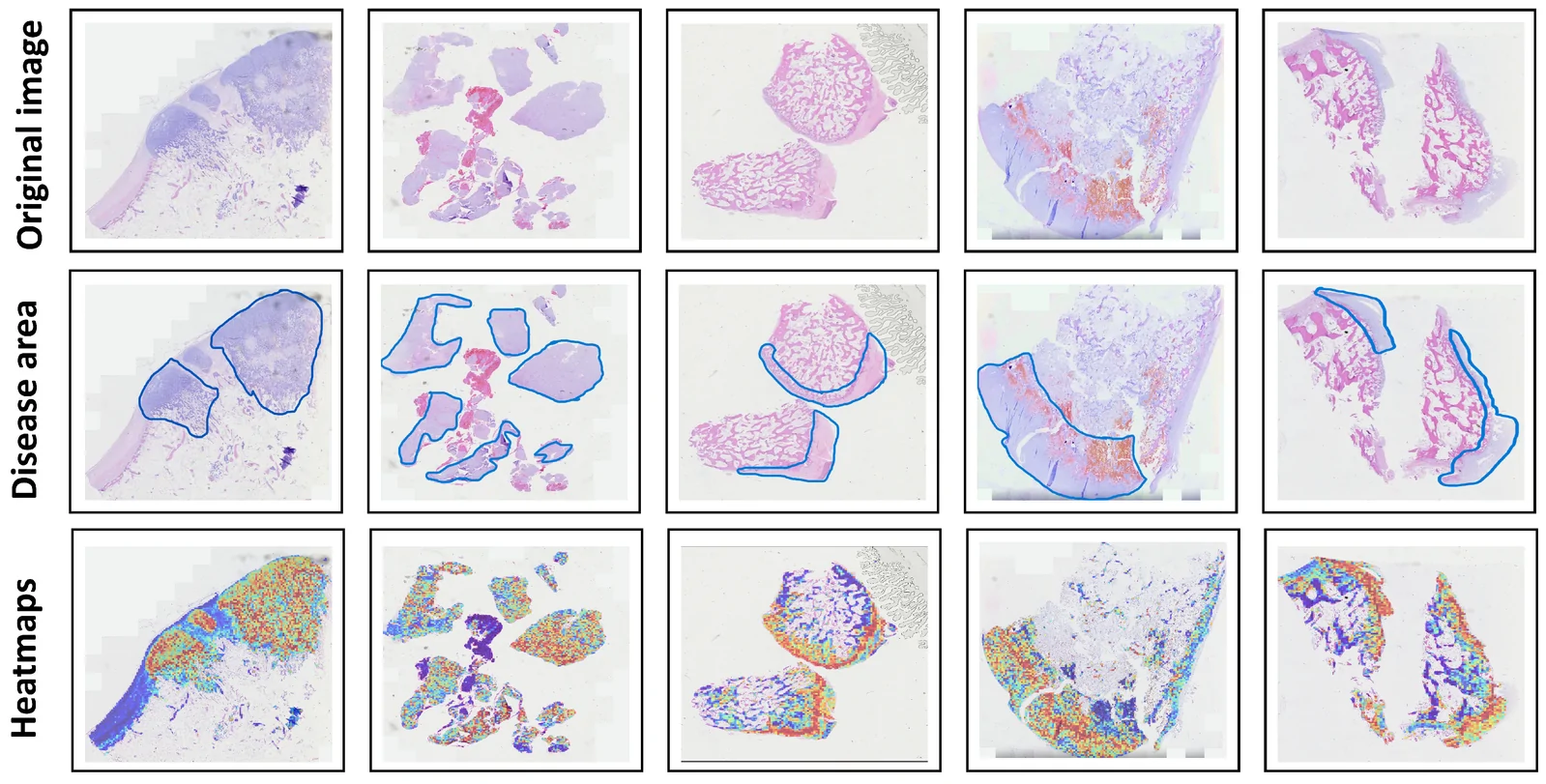
Heatmap visualization and clinical consistency analysis of XChondNet on representative chondrogenic tumor cases. The top row shows the original pathological images, the middle row shows the pathologist-annotated lesion regions, and the bottom row shows the heatmap-based recognition results generated by XChondNet. This visualization is used to assess whether the model-highlighted regions are spatially consistent with clinically meaningful lesion areas identified by the pathologist.

As shown in Figure 11, the heatmaps provide intuitive evidence that XChondNet can focus on diagnostically meaningful regions. The images in columns 1, 4, and 5 show heatmap visualizations of osteochondroma. Taking the first column as an example, the tumor presents a typical three-layer spatial structure, including the fibrous membrane, cartilage layer, and bone layer. The second column shows chondromyxoid fibroma, which is usually lobulated with dense perilobular cells and relatively low cellular density in the center. The third column shows subungual exostosis, which has certain histological similarity to osteochondroma and also presents a layered spatial structure, gradually transitioning from the peripheral fibrous layer composed of spindle cells to the germinative fibroblast layer, cartilage layer, and cartilage osteogenic layer. The high-attention regions of XChondNet showed clear spatial correspondence with the pathologist-annotated lesion regions, suggesting that the model can perceive important spatial structural information in chondrogenic tumors.

To further provide quantitative evidence beyond visual interpretation, we conducted an IoU-based overlap analysis between the model-generated heatmaps and pathologist-annotated ROIs. The heatmaps were normalized and binarized to obtain model-highlighted high-attention regions, while the pathologist-annotated ROIs were used as expert reference regions. The IoU values were used to evaluate the spatial consistency between model attention and expert annotation.

**Figure 12:**
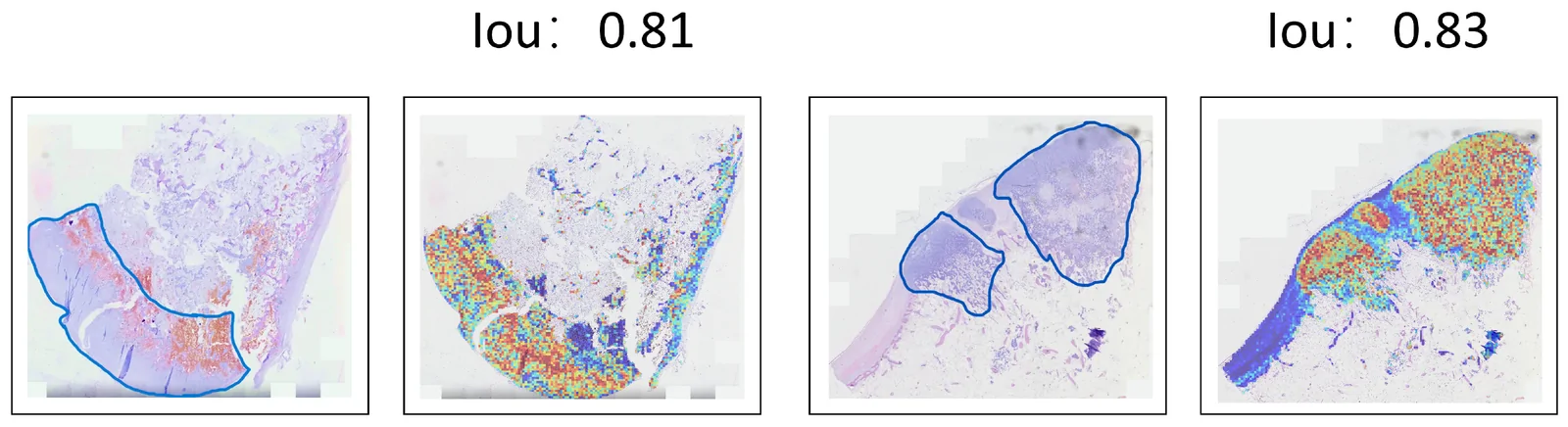
Representative examples of IoU-based consistency analysis between XChondNet heatmaps and pathologist-annotated ROIs. The blue contours indicate the pathologist-annotated ROIs, and the heatmaps represent model-highlighted discriminative regions. The IoU values are 0.81 and 0.83, respectively.

As shown in Figure 12, the model-highlighted regions showed substantial overlap with pathologist-annotated ROIs, with IoU values of 0.81 and 0.83 in the representative cases. These results indicate that the heatmaps generated by XChondNet are not only visually interpretable, but also quantitatively consistent with expert-defined diagnostic regions.

To further assess the consistency and reproducibility of the spatial correspondence between model attention and expert-defined diagnostic regions, we extended the IoU analysis to the entire held-out test cohort. Three pathologists with different levels of diagnostic experience independently annotated the diagnostically relevant ROIs. For each test case, the IoU between the binarized high-attention regions generated by XChondNet and the corresponding pathologist-annotated ROIs was calculated. Across the entire test cohort, the mean IoU was 0.73±0.11 for the less-experienced pathologist, 0.78±0.09 for the experienced pathologist, and 0.82±0.08 for the highly experienced pathologist, respectively, with an overall mean IoU of 0.77 across the three pathologists. The IoU showed a gradual increase with the level of diagnostic expertise, indicating stronger spatial correspondence between the model-highlighted regions and the annotations provided by more experienced pathologists. In addition, inter-observer agreement among the three pathologists was evaluated to assess the consistency of expert annotations. These cohort-level results provide quantitative evidence that the spatial regions highlighted by XChondNet show consistent correspondence with expert-defined diagnostic regions across the test cohort, rather than being limited to selected representative cases.

### 4.10. Computational Complexity and Hyperparameter Settings

To further evaluate the practical feasibility and reproducibility of XChondNet, we added computational complexity analysis and summarized the main hyperparameters used in the experiments. The computational cost was evaluated in terms of trainable parameters, inference time, and peak GPU memory usage. All measurements were conducted on an NVIDIA RTX 3090 GPU (NVIDIA Corporation, Santa Clara, CA, USA) under the same experimental environment.

As shown in Table 11, XChondNet maintains acceptable computational cost during WSI-level inference. The reported inference time and GPU memory usage provide a practical reference for evaluating the deployment feasibility of the proposed method in computational pathology scenarios.

As shown in Table 12, the key experimental settings and training hyperparameters were explicitly reported to improve the reproducibility of XChondNet. These settings were kept consistent across different feature extractors and comparative experiments.

## 5. Conclusions

In this study, we developed and validated XChondNet, an explainable computational pathology model for chondrogenic tumor diagnosis. By integrating pathological and positional features with a parallel classifier mechanism, XChondNet effectively captures spatial structural information and improves the reliability of WSI-based chondrogenic tumor classification. Experimental results demonstrated that XChondNet consistently achieved strong performance across different feature extractors. In particular, compared with two strong MIL baselines, DTFD-MIL and RRT-MIL, XChondNet improved the average ACC from 85.16% to 86.58%, corresponding to a 1.42 percentage-point improvement. The improvement was statistically significant, as confirmed by both a paired *t*-test ($p=2.61\times 10^{-5}$) and a Wilcoxon signed-rank test (p=0.0078), providing statistical evidence for the performance advantage of XChondNet. Furthermore, the attention maps generated by XChondNet highlighted regions consistent with pathologists’ diagnostic concerns, demonstrating its potential to provide interpretable evidence for AI-assisted diagnosis.

To further assess the reliability of the observed performance improvement, we performed a case-level bootstrap analysis to estimate the 95% confidence interval of the ACC difference between XChondNet and DTFD-MIL. XChondNet achieved a 1.42 percentage-point improvement in average ACC, with a 95% confidence interval of [0.4%, 2.5%]. Since the confidence interval did not include zero, this analysis provides additional evidence supporting the observed performance improvement. Nevertheless, the relatively limited sample size and single-center nature of the current cohort may constrain the generalizability of the findings. In future work, we will incorporate additional cases to further increase the sample size and diversity of the cohort, and will conduct validation on larger and independently collected datasets. These efforts will provide more comprehensive evidence for the reliability, generalizability, and potential clinical applicability of XChondNet.

Despite these promising results, this study has several limitations, including variations in staining styles and the relatively limited number of available samples. In future work, we will explore multimodal technologies to further improve spatial feature representation and diagnostic robustness. In addition, generative techniques will be investigated to alleviate the limitation of insufficient training samples and improve model generalization. Integrating imaging and pathological image generation technologies may provide additional representative samples from limited patient cohorts and further enhance the performance of computer-aided diagnosis. Overall, XChondNet provides a promising solution for explainable chondrogenic tumor classification and demonstrates the potential of spatial-context-aware computational pathology for supporting pathological diagnosis. Future studies involving larger, more diverse, and independent datasets will be necessary to further validate its clinical applicability.

## Figures and Tables

**Table 1:** Pathological data type distribution and data division.

Category	Data Type		Data Division $\left(8:1:1\right)$		
	WSI	ROI	Train	Valid	Test
OC	31	295	235	30	30
ED	17	159	117	17	25
CF	39	507	412	61	34
SE	15	107	76	17	14
CB	15	216	168	21	27
CD	22	188	139	16	33

**Table 2:** Comparative performance of different MIL-based methods on Conch, Resnet-50, UNI, and PLIP feature extractors. The best results are highlighted in **bold**. Evaluation metrics include ACC, AUC, F1 score, and Recall.

Method	Conch				Resnet-50			
	ACC (%)	AUC (%)	F1 (%)	Recall (%)	ACC (%)	AUC (%)	F1 (%)	Recall (%)
MIL	68.67 ± 2.29	85.52 ± 0.98	68.86 ± 2.27	68.67 ± 2.29	58.17 ± 2.38	77.23 ± 1.23	58.86 ± 2.57	58.79 ± 2.47
Max-MIL	77.39 ± 2.13	92.12 ± 1.11	77.86 ± 2.12	77.47 ± 2.11	71.27 ± 2.46	88.39 ± 1.44	71.83 ± 2.19	71.82 ± 2.13
Mean-MIL	76.89 ± 3.07	93.67 ± 1.80	76.48 ± 2.98	76.26 ± 3.03	70.47 ± 2.47	87.98 ± 1.83	70.67 ± 2.31	70.66 ± 2.42
Att-MIL	82.37 ± 2.49	96.62 ± 0.81	82.48 ± 2.31	82.44 ± 2.46	77.94 ± 2.58	94.62 ± 1.28	77.91 ± 2.49	77.89 ± 2.51
Gatt-MIL	83.14 ± 1.94	97.02 ± 1.12	83.46 ± 1.89	83.48 ± 1.91	79.18 ± 2.04	95.06 ± 1.23	78.48 ± 2.18	78.66 ± 2.24
CLAM-SB	82.07 ± 2.34	96.04 ± 0.96	82.24 ± 2.14	82.03 ± 2.38	79.66 ± 2.67	94.51 ± 0.61	79.46 ± 2.81	79.83 ± 2.88
CLAM-MB	83.83 ± 2.23	96.83 ± 0.94	84.01 ± 2.02	83.77 ± 2.13	80.25 ± 2.20	94.18 ± 0.65	80.29 ± 2.10	80.22 ± 2.14
AB-MIL	83.77 ± 1.88	96.23 ± 0.77	83.94 ± 1.91	83.77 ± 1.91	80.75 ± 1.91	95.14 ± 1.12	80.56 ± 2.01	80.46 ± 1.97
MHIM-MIL	84.65 ± 1.86	97.09 ± 0.20	84.80 ± 1.47	84.65 ± 1.86	81.23 ± 1.97	95.22 ± 1.37	81.86 ± 1.82	81.33 ± 1.91
TRANS-MIL	84.92 ± 1.74	96.64 ± 0.64	84.99 ± 1.61	84.92 ± 1.74	81.09 ± 1.74	94.53 ± 1.13	81.03 ± 1.73	81.03 ± 1.76
DS-MIL	84.78 ± 1.40	97.23 ± 0.20	84.94 ± 1.30	84.78 ± 1.40	82.04 ± 1.12	95.88 ± 0.89	82.00 ± 1.07	81.96 ± 1.04
DTFD-MIL	85.89 ± 1.31	96.89 ± 0.56	85.86 ± 1.37	85.87 ± 1.32	83.52 ± 1.10	95.10 ± 1.71	83.22 ± 1.38	83.35 ± 1.41
RRT-MIL	85.46 ± 1.21	**97.67 ± 0.53**	85.53 ± 1.61	85.46 ± 1.21	83.55 ± 1.72	95.78 ± 0.76	82.47 ± 1.62	82.42 ± 1.61
**XChondNet**	**87.09 ± 1.38**	97.26 ± 0.51	**87.14 ± 1.44**	**87.08 ± 1.36**	**84.71 ± 2.16**	**95.96 ± 0.91**	**84.69 ± 2.57**	**84.72 ± 2.59**
**Method**	**UNI**				**PLIP**			
	**ACC (%)**	**AUC (%)**	**F1 (%)**	**Recall (%)**	**ACC (%)**	**AUC (%)**	**F1 (%)**	**Recall (%)**
MIL	69.21 ± 2.08	86.41 ± 1.72	69.63 ± 2.21	69.67 ± 2.31	64.27 ± 2.91	82.23 ± 1.64	63.76 ± 2.62	63.77 ± 2.61
Max-MIL	79.41 ± 1.83	93.25 ± 1.07	79.75 ± 1.92	79.71 ± 1.94	74.37 ± 2.26	91.29 ± 1.67	74.33 ± 2.17	74.32 ± 2.18
Mean-MIL	78.91 ± 1.73	93.37 ± 1.16	78.87 ± 1.77	78.89 ± 1.76	74.71 ± 2.41	92.31 ± 1.72	74.36 ± 2.04	74.38 ± 2.03
Att-MIL	83.47 ± 2.17	96.53 ± 0.97	83.46 ± 2.11	83.44 ± 2.12	80.74 ± 2.46	95.27 ± 1.09	80.91 ± 2.38	80.96 ± 2.41
Gatt-MIL	83.87 ± 1.97	96.31 ± 1.12	83.91 ± 1.89	83.93 ± 1.92	80.89 ± 2.32	95.41 ± 0.96	80.78 ± 2.41	80.77 ± 2.43
CLAM-SB	83.01 ± 2.43	96.49 ± 1.23	83.04 ± 2.47	83.06 ± 2.48	80.46 ± 2.52	95.71 ± 0.88	80.51 ± 2.53	80.53 ± 2.54
CLAM-MB	84.21 ± 1.78	97.02 ± 0.77	84.06 ± 1.81	84.05 ± 1.83	81.27 ± 2.21	96.21 ± 0.92	81.31 ± 2.23	81.26 ± 2.31
AB-MIL	84.01 ± 1.66	96.89 ± 0.87	84.07 ± 1.95	84.08 ± 1.98	81.79 ± 1.98	96.41 ± 0.88	81.81 ± 1.93	81.86 ± 1.89
MHIM-MIL	85.25 ± 1.67	97.16 ± 0.44	85.30 ± 1.69	85.35 ± 1.66	83.31 ± 1.77	96.87 ± 1.12	83.46 ± 1.81	82.43 ± 1.87
TRANS-MIL	83.82 ± 1.94	96.44 ± 1.14	83.89 ± 1.97	83.87 ± 1.91	82.78 ± 1.81	95.53 ± 0.87	82.83 ± 1.79	82.84 ± 1.82
DS-MIL	85.82 ± 1.39	97.01 ± 0.56	85.80 ± 1.38	85.85 ± 1.41	84.27 ± 1.61	96.49 ± 1.03	84.26 ± 1.59	84.23 ± 1.57
DTFD-MIL	86.21 ± 0.97	96.96 ± 0.71	86.30 ± 1.03	86.34 ± 1.06	84.30 ± 1.87	96.38 ± 1.11	84.36 ± 1.84	84.43 ± 1.77
RRT-MIL	86.82 ± 0.83	**97.81 ± 0.41**	86.93 ± 0.74	86.88 ± 0.76	85.51 ± 1.63	96.21 ± 0.67	85.53 ± 1.67	85.56 ± 1.71
**XChondNet**	**88.03 ± 0.97**	97.41 ± 0.84	**88.14 ± 1.02**	**88.18 ± 1.01**	**86.49 ± 1.94**	**96.35 ± 0.84**	**86.63 ± 1.87**	**86.49 ± 1.91**

**Table 3:** Ablation results of the proposed model under different feature extractors (Conch, ResNet-50, UNI, and PLIP), evaluated by ACC, AUC, F1, and Recall. The best results are highlighted in **bold**.

Method	Conch				Resnet-50			
	ACC (%)	AUC (%)	F1 (%)	Recall (%)	ACC (%)	AUC (%)	F1 (%)	Recall (%)
w/o pos	83.67 ± 2.12	95.32 ± 0.98	83.72 ± 2.21	83.61 ± 2.19	81.36 ± 2.84	94.72 ± 1.12	81.41 ± 2.93	81.28 ± 2.89
w/o Intra	84.12 ± 1.96	95.64 ± 1.02	84.25 ± 2.01	84.09 ± 1.94	82.07 ± 2.55	95.01 ± 1.04	82.15 ± 2.61	82.03 ± 2.58
w/o Inter	85.03 ± 1.77	96.21 ± 0.89	85.11 ± 1.82	85.07 ± 1.85	83.14 ± 2.33	95.32 ± 0.97	83.19 ± 2.41	83.11 ± 2.39
w/o Pro	85.46 ± 2.08	96.42 ± 0.95	85.39 ± 2.12	85.44 ± 2.05	83.42 ± 2.47	95.48 ± 0.95	83.39 ± 2.52	83.44 ± 2.49
Ours	**87.09 ± 1.38**	**97.26 ± 0.51**	**87.14 ± 1.44**	**87.08 ± 1.36**	**84.71 ± 2.16**	**95.96 ± 0.91**	**84.69 ± 2.57**	**84.72 ± 2.59**
**Method**	**UNI**				**PLIP**			
	**ACC (%)**	**AUC (%)**	**F1 (%)**	**Recall (%)**	**ACC (%)**	**AUC (%)**	**F1 (%)**	**Recall (%)**
w/o pos	84.92 ± 1.62	95.86 ± 1.07	85.01 ± 1.71	84.97 ± 1.69	83.41 ± 2.45	94.92 ± 1.03	83.56 ± 2.52	83.39 ± 2.48
w/o Intra	85.67 ± 1.48	96.12 ± 0.99	85.74 ± 1.55	85.69 ± 1.53	84.27 ± 2.21	95.14 ± 0.97	84.36 ± 2.28	84.25 ± 2.24
w/o Inter	86.42 ± 1.25	96.58 ± 0.91	86.49 ± 1.31	86.44 ± 1.29	85.06 ± 2.08	95.63 ± 0.91	85.19 ± 2.13	85.11 ± 2.12
w/o Pro	86.71 ± 1.34	96.72 ± 0.88	86.77 ± 1.39	86.73 ± 1.36	85.32 ± 2.14	95.78 ± 0.89	85.41 ± 2.18	85.36 ± 2.15
Ours	**88.03 ± 0.97**	**97.41 ± 0.84**	**88.14 ± 1.02**	**88.18 ± 1.01**	**86.49 ± 1.94**	**96.35 ± 0.84**	**86.63 ± 1.87**	**86.49 ± 1.91**

**Table 4:** Ablation results of the proposed model about the parallel classifier mechanism under different feature extractors (Conch, ResNet-50, UNI, and PLIP), evaluated by ACC, AUC, F1, and Recall. The best results are highlighted in **bold**.

Setting	Performance			
	ACC (%)	AUC (%)	F1 Score (%)	Recall (%)
Conch (**✓**)	**87.09 ± 1.38**	**97.26 ± 0.51**	**87.14 ± 1.44**	**87.08 ± 1.36**
Conch (**✗**)	85.72 ± 1.65	95.84 ± 0.73	85.61 ± 1.58	85.47 ± 1.62
Resnet50 (**✓**)	**84.71 ± 2.16**	**95.96 ± 0.91**	**84.69 ± 2.57**	**84.72 ± 2.59**
Resnet50 (**✗**)	82.94 ± 2.43	94.87 ± 1.12	82.68 ± 2.71	82.73 ± 2.65
UNI (**✓**)	**88.03 ± 0.97**	**97.41 ± 0.84**	**88.14 ± 1.02**	**88.18 ± 1.01**
UNI (**✗**)	86.42 ± 1.28	96.35 ± 1.07	86.51 ± 1.33	86.47 ± 1.29
PLIP (**✓**)	**86.49 ± 1.94**	**96.35 ± 0.84**	**86.63 ± 1.87**	**86.49 ± 1.91**
PLIP (**✗**)	84.92 ± 2.21	95.12 ± 1.03	85.01 ± 2.14	84.88 ± 2.19

**Table 5:** Temporal validation results on the chronologically separated test set. Cases collected from 2017 to 2022 were used for training and validation, while cases collected from 2023 to 2024 were held out as an independent temporal test set.

Method	Feature Extractor	ACC (%)	AUC (%)	F1 (%)	Recall (%)
DTFD-MIL	UNI	85.47 ± 0.46	95.87 ± 0.82	85.42 ± 1.69	85.38 ± 1.41
RRT-MIL	UNI	85.93 ± 0.66	97.12 ± 0.75	85.90 ± 0.68	85.83 ± 0.62
**XChondNet**	UNI	**86.82 ± 1.01**	**97.23 ± 0.99**	**86.87 ± 1.41**	**86.76 ± 1.38**

**Table 6:** Domain-shift robustness analysis under different feature-domain settings. ACC values are reported across four feature extractors.

Method	Conch	ResNet-50	UNI	PLIP	Mean ACC
DTFD-MIL	85.89	83.52	86.21	84.30	84.98
RRT-MIL	85.46	83.55	86.82	85.51	85.34
**XChondNet**	**87.09**	**84.71**	**88.03**	**86.49**	**86.58**

**Table 7:** Final comparison with 95% confidence intervals under the UNI feature extractor.

Method	ACC (%)	AUC (%)	F1 (%)	Recall (%)
DTFD-MIL	86.21 [85.01, 87.41]	96.96 [96.08, 97.84]	86.30 [85.02, 87.58]	86.34 [85.02, 87.66]
RRT-MIL	86.82 [85.79, 87.85]	**97.81 [97.30, 98.32]**	86.93 [86.01, 87.85]	86.88 [85.94, 87.82]
**XChondNet**	**88.03 [86.83, 89.23]**	97.41 [96.37, 98.45]	**88.14 [86.87, 89.41]**	**88.18 [86.93, 89.43]**

**Table 8:** Statistical significance analysis of ACC improvements between XChondNet and strong MIL baselines across four feature-domain settings. ** indicates p<0.01; *** indicates p<0.001.

Comparison	Mean ACC Difference (%)	Paired *t*-Test	Wilcoxon Test	Significance
XChondNet vs. DTFD-MIL	1.60	p=0.0074	p=0.1250	**
XChondNet vs. RRT-MIL	1.25	p=0.0028	p=0.1250	**
XChondNet vs. DTFD-MIL and RRT-MIL	1.42	$p=2.61\times 10^{-5}$	p=0.0078	***

**Table 9:** Bootstrap-based confidence intervals of XChondNet under the UNI feature extractor.

Metric	Observed Value (%)	Bootstrap 95% CI (%)
ACC	88.03	[85.76, 90.18]
AUC	97.41	[96.23, 98.47]
F1 score	88.14	[85.92, 90.37]
Recall	88.18	[85.69, 90.44]

**Table 10:** Per-class performance of XChondNet under the UNI feature extractor.

Class	Precision (%)	Recall (%)	F1 Score (%)	AUC (%)
OC	89.95	88.70	89.32	98.21
ED	87.40	88.60	88.00	97.36
CF	87.90	87.24	87.57	97.48
SE	86.55	88.87	87.69	96.62
CB	90.20	89.10	89.65	98.07
CD	86.77	86.57	86.67	96.72

**Table 11:** Computational complexity analysis of XChondNet.

Model	Params (M)	Inference Time (s/WSI)	GPU Memory (GB)
XChondNet	2.61	7.94	7.92

**Table 12:** Main hyperparameters used in XChondNet.

Hyperparameter	Value
Magnification	20×
Feature extractors	Conch, ResNet-50, UNI, PLIP
Feature dimension	512
Optimizer	Adam
Learning rate	0.001
Weight decay	$1\times 10^{-5}$
Batch size	1
Validation interval	Every 200 epochs
Early stopping	Yes
Evaluation protocol	5-fold cross-validation
Train/Validation/Test split	8:1:1
Number of classes	6
GPU	NVIDIA RTX 3090

## Data Availability

The data are not publicly available due to privacy and ethical restrictions.
